# Selective and green conversion of 5-HMF to FDCA *via* enzymatic (laccase) and transition metal (MnO_2_ and Co–Mn/AC) catalysis in an integrated system

**DOI:** 10.1039/d5ra04438c

**Published:** 2025-09-08

**Authors:** Rakesh J. Gujar, Raju T. Thombe, Pratibha Dhindale

**Affiliations:** a Department of Chemical Engineering and Bioprocess Technology, Institute of Chemical Technology (ICT) Mumbai Maharashtra 400019 India rakeshgujar0898@gmail.com rajuthombe@gmail.com; b Department of Chemical Engineering and Green Technology, Institute of Chemical Technology (ICT) Mumbai Maharashtra 400019 India pratibhadhindale19@gmail.com

## Abstract

The sustainable synthesis of bio-based monomers from renewable biomass intermediates is a central goal in green chemistry and biorefinery innovation. This study introduces a synergistic catalytic–enzymatic strategy for the efficient and eco-friendly oxidation of 5-hydroxymethylfurfural (5-HMF) into 2,5-furandicarboxylic acid (FDCA), a key monomer for next-generation biodegradable plastics. The catalytic phase employed non-noble metal catalysts, MnO_2_ and Co–Mn supported on activated carbon (Co–Mn/AC), under mild batch reaction conditions at 90 °C. Through systematic optimization, a metal-modified catalyst composition was developed to enhance both conversion and selectivity. Complementing this, the enzymatic oxidation step utilized laccase, a sustainable biocatalyst, immobilized in a packed-bed column reactor operating under continuous flow. Preliminary batch studies were conducted to understand the conversion kinetics and establish optimal parameters. Maximum FDCA yield was achieved with an enzyme concentration of 1 mg mL^−1^ at 40 °C and pH 5. Additionally, the influence of substrate concentration, residence time, and reaction temperature was assessed for process intensification. This dual-step process exemplifies a green and scalable pathway, merging heterogeneous catalysis and biocatalysis for the valorization of biomass into high-value bio-based chemicals. The approach provides a forward-looking model for industrial adoption of sustainable oxidation technologies in the development of environmentally friendly polymers.

## Abstract 2

The sustainable synthesis of bio-based monomers from renewable biomass intermediates is a central goal in green chemistry and biorefinery innovation.

## Graphical abstract

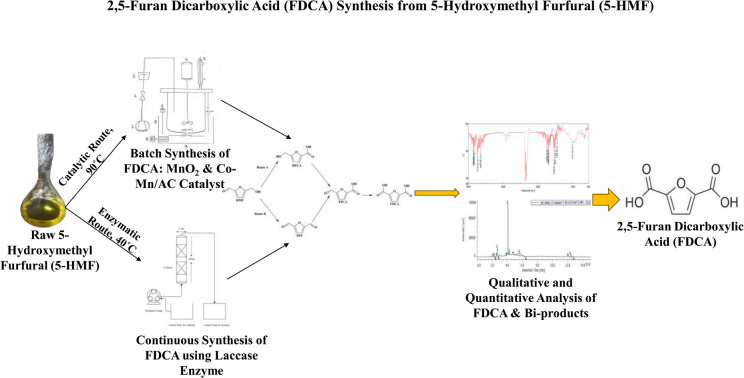

## 1 Introduction

The growing demand for environmentally sustainable chemical processes, driven by the global shift away from fossil-based resources, has intensified interest in biomass-derived platform molecules for industrial applications.^1^ One such molecule, 5-hydroxymethylfurfural (5-HMF), has emerged as a key intermediate due to its versatile chemical structure and ability to be converted into a variety of high-value derivatives, such as 2,5-diformylfuran (DFF), 5-formyl-2-furancarboxylic acid (FFCA), 5-hydroxymethyl-2-furancarboxylic acid (HMFCA), and most notably, 2,5-furandicarboxylic acid (FDCA).^2,3^ Among these, FDCA has gained significant attention as a renewable monomer for the production of polyethylene furanoate (PEF), a sustainable alternative to conventional polyethylene terephthalate (PET). PEF not only offers enhanced thermal and barrier properties but also aligns with circular economy goals, making FDCA a critical building block for future bio-based plastics.^4,5^

**Fig. 1:**
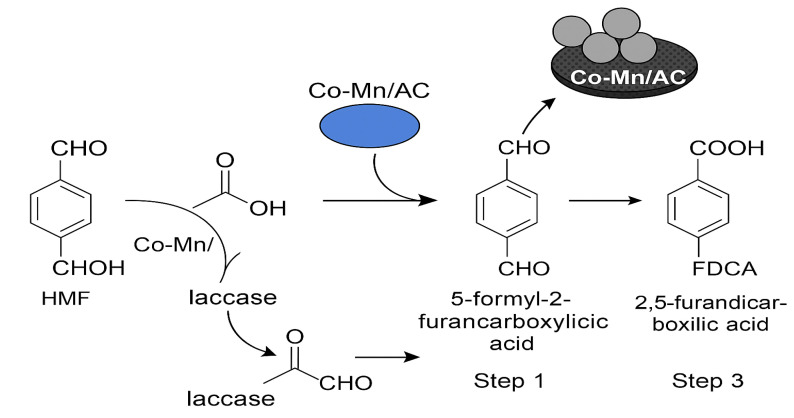
Proposed reaction mechanism for the oxidation of HMF to FDCA using a hybrid Co–Mn/AC-laccase catalyst system.

Recognizing its potential, the U.S. Department of Energy has identified 2,5-furandicarboxylic acid (FDCA) as one of the top twelve bio-based platform chemicals with significant industrial relevance. Structurally, FDCA possesses two carboxylic acid functional groups, which are responsible for its high thermal stability (melting point: 342 °C) and chemical reactivity, making it a valuable precursor for the synthesis of bio-based polymers such as polyethylene furanoate (PEF). However, despite its promising applications, the selective and efficient oxidation of 5-hydroxymethylfurfural (5-HMF) to FDCA under environmentally benign and economically feasible conditions remains a critical challenge in green chemistry. Fig. 1 illustrates the mechanistic pathway for the oxidation of 5-HMF to FDCA, highlighting the intermediate formation of 5-hydroxymethyl-2-furancarboxylic acid (HMFCA), 5-formyl-2-furancarboxylic acid (FFCA), and 2,5-diformylfuran (DFF) as key steps in the overall transformation.

This bottleneck continues to limit the scalability and commercial viability of biomass valorization routes for FDCA production.^6^ Recent research has increasingly focused on a variety of catalytic systems, not only using noble metals but also emphasizing more cost-effective non-noble metal catalysts such as manganese dioxide (MnO_2_), cobalt–manganese on activated carbon (Co–Mn/AC), copper–manganese oxides (Cu–MnO_*x*_), and nickel-based formulations. These catalysts have shown promise in facilitating aerobic oxidation reactions under milder or solvent-assisted conditions while maintaining economic viability.^7^ Among catalytic strategies, systems employing non-noble metal catalysts, notably MnO_2_ and Co–Mn/activated carbon (Co–Mn/AC), have demonstrated improved selectivity and economic viability, especially when operated under solvent-assisted conditions using air or molecular oxygen as the oxidant. Despite the promise of catalytic oxidation methods for converting 5-HMF to FDCA, many of these processes operate under harsh conditions, including elevated temperatures and pressures. These conditions often lead to the formation of undesired byproducts, which compromise both yield and environmental sustainability of the overall process.^8,9^ In contrast, enzymatic oxidation has gained attention as a greener alternative, offering advantages such as mild operating conditions, high substrate specificity, and minimal side product formation. Among the oxidative enzymes investigated, laccase has emerged as a particularly promising biocatalyst for selective oxidation reactions due to its ability to oxidize a wide range of phenolic and non-phenolic substrates using molecular oxygen as the terminal oxidant.^10^ Among the oxidative enzymes explored, laccase has emerged as a particularly effective biocatalyst. It enables the stepwise oxidation of 5-HMF through well-defined intermediates, including 5-hydroxymethyl-2-furancarboxylic acid (HMFCA) and 5-formyl-2-furancarboxylic acid (FFCA), culminating in the formation of FDCA. This biocatalytic conversion is significantly enhanced in the presence of suitable redox mediators, which facilitate electron transfer and broaden the oxidative capacity of the enzyme. The laccase-mediated pathway thus represents a compelling route for the green and selective synthesis of FDCA from renewable feedstocks.^11,12^ To enhance the feasibility of enzymatic processes at an industrial scale, recent developments have employed immobilized laccase within packed-bed reactors. These continuous biocatalytic systems offer advantages such as improved operational stability, ease of product separation, enhanced enzyme reusability, and reduced downstream processing costs. This study presents an integrated overview of both chemical and enzymatic oxidation strategies and proposes a dual-pathway approach comprising a batch-mode catalytic route using non-noble metal catalysts, alongside a continuous packed-bed system employing immobilized laccase. The influence of process variables, including reaction time, temperature, pH, and substrate concentration, was systematically studied to optimize conversion efficiency and yield, supporting the development of scalable and sustainable FDCA production technologies, conditions, eco-friendliness, and potential for integration into continuous biocatalytic systems, laccase presents a promising alternative to conventional chemical oxidants in green synthesis pathways.^13–15^

To enhance the practicality of enzymatic oxidation for industrial applications, immobilized enzyme reactors, such as packed-bed columns, are increasingly being adopted.^16^ These systems provide several advantages, including improved enzyme stability, ease of separation and reuse, and reduced operational costs, making them well-suited for continuous flow bioprocesses.^17^ Moreover, achieving high product yields and process efficiency depends critically on the optimization of parameters such as reaction time, temperature, pH, and substrate concentration.

**Fig. 2:**
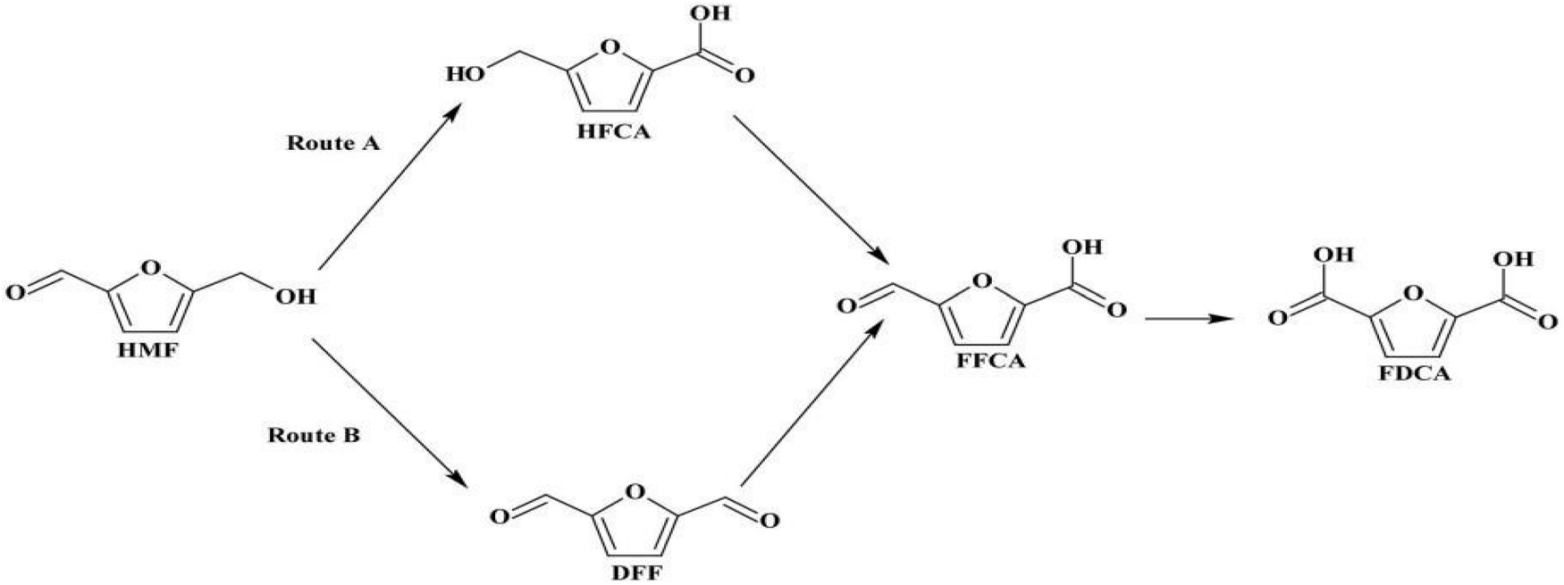
Oxidative conversion pathways of HMF to FDCA *via* HFCA, DFF, and FFCA intermediates.

In this context, the present study explores a dual-pathway strategy for the oxidative conversion of 5-HMF to FDCA (Fig. 2). The first pathway involves the use of a non-noble metal catalyst for the batch-mode oxidation of 5-HMF, offering a cost-effective and robust chemical route.^18^ The second pathway employs immobilized laccase within a continuous packed-bed reactor, enabling enzymatic oxidation under mild and environmentally benign conditions.^19,20^ Reaction kinetics and the influence of key process variables were systematically studied to identify the optimal conditions for maximum FDCA yield.^21^ This integrated catalytic-biotechnological approach presents a promising framework for the green and scalable production of FDCA, aligning with broader objectives of circular chemistry and a sustainable bioeconomy.^22,23^ The rationale for combining laccase with Co–Mn/AC lies in leveraging the strengths of both systems. Laccase offers high selectivity under mild, eco-friendly conditions but has limited oxidation power for some substrates. Co–Mn/AC enhances redox activity and broadens the substrate range. Together, this hybrid system improves overall efficiency, selectivity, and reusability—while also lowering costs and making the process more scalable compared to single systems. This integrated approach addresses the key limitations of using either catalyst alone. This study presents a hybrid system that combines the high redox activity of Co–Mn/AC with the selectivity and mild conditions of immobilized laccase. This integration offers multiple benefits:

(1) Fewer process steps due to seamless oxidation transitions.

(2) Higher yield and selectivity, leveraging enzyme specificity.

(3) Lower energy requirements with mild reaction conditions.

(4) Cost-effective, avoiding noble metals and harsh reagents.

(5) Scalable, using continuous packed-bed enzyme reactors.

Together, this dual system overcomes key limitations of individual methods, offering a greener, efficient, and industry-relevant route for FDCA production.

## 2 Materials and methods

5-HMF obtained from in-house research conducted by a colleague was used in this study. The samples were transferred to a glass bottle and stored at −20 °C in a deep freezer to enhance stability and prevent cross-contamination. All chemicals and reagents used were of analytical or HPLC grade. Acetonitrile and methanol (HPLC grade) were procured from Molychem, Mumbai, India. Sodium acetate, standard-grade TEMPO, and 2,5-furandicarboxylic acid (FDCA) were sourced from Zeta Scientific. Laccase enzyme, used for the enzymatic oxidation process, was obtained from Sigma-Aldrich. Aminopropyl triethoxy silane (APTES), and guaiacol were supplied by S.D. Fine-Chem Limited. Additional reagents, including NaH_2_PO_4_, Na_2_HPO_4_, hydrochloric acid (HCl), hydrofluoric acid (HF), and sodium hydroxide (NaOH), were purchased from Himedia Laboratories Pvt. Ltd, Mumbai, India. Manganese dioxide (MnO_2_) and cobalt–manganese supported on activated carbon (Co–Mn/AC) (Co-7 wt%, Mn-3 wt%, AC-90 wt%) used as heterogeneous catalysts for the oxidation of 5-HMF were also sourced from Himedia.

### 2.1 Methods

#### 2.1.1 Laccase immobilization method

##### 2.1.1.1 Physicochemical cleaning and pre-activation of glass beads

Glass beads were rigorously cleaned through multiple sequential rinses with deionized (DI) water (3–4 cycles) to effectively remove surface-adhered particulates, residual chemical agents, and organic contaminants. Following the washing procedure, the beads were dried in a hot-air oven under controlled temperature conditions ranging between 35 °C and 40 °C to ensure the complete elimination of residual surface moisture. This drying step was essential for preserving the physicochemical integrity and surface reactivity of the glass beads, thereby ensuring their suitability for subsequent chemical functionalization and modification processes.^24^

##### 2.1.1.2 Surface functionalization of glass beads *via* HF etching

To initiate surface activation, pre-cleaned glass beads were packed into an acid-resistant, vertically aligned glass column. A 4% (v/v) hydrofluoric acid (HF) solution was then prepared by cautiously diluting concentrated HF with deionized water inside a fume hood, in accordance with established safety protocols for handling highly corrosive substances. The prepared HF solution was recirculated through the column for 5 hours using a peristaltic pump, promoting consistent and controlled surface etching of the glass beads. This etching step effectively enhanced surface roughness and increased the concentration of silanol (Si–OH) functional groups, which are essential for subsequent surface modifications. To verify the increase in silanol groups resulting from HF treatment, Fourier-transform infrared spectroscopy (FTIR) analysis was conducted both before and after etching. Prior to HF treatment, FTIR spectra showed weak or minimal absorbance in the characteristic Si–OH stretching region (∼3300–3700 cm^−1^), indicating a relatively low surface hydroxyl concentration. Following HF etching, a pronounced increase in absorption intensity within this range was observed, confirming the formation and enrichment of Si–OH functional groups on the glass bead surface. This spectral evidence validated the effective surface activation achieved through controlled acid etching.

After etching, the beads were subjected to extensive rinsing with DI water (≥5 cycles) to completely eliminate any residual HF and ensure the biocompatibility of the surfaces for downstream enzymatic applications.^25^

##### 2.1.1.3 Amino-functionalization of glass beads using APTES

**Fig. 3:**
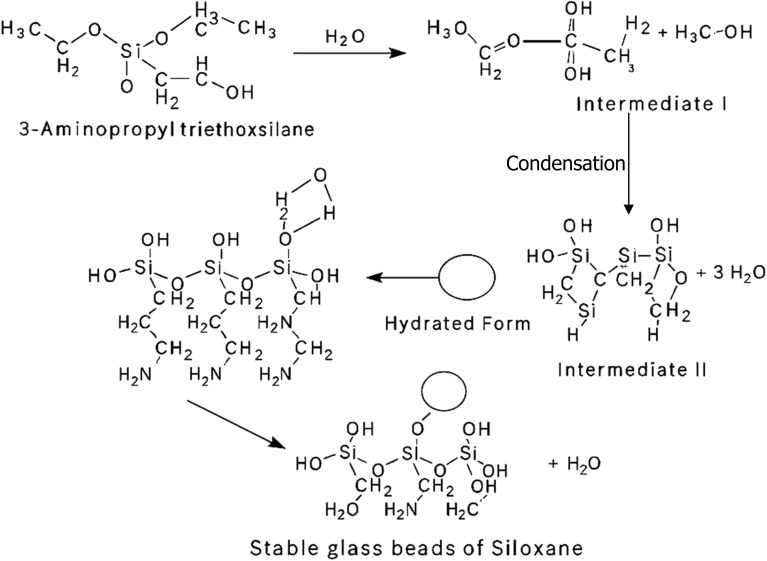
Schematic illustration of glass bead surface functionalization using 3-aminopropyltriethoxysilane (APTES).

The HF-etched glass beads were subsequently functionalized with amino groups by circulating a 5% (v/v) solution of 3-aminopropyltriethoxysilane (APTES) in toluene through the packed column at 90 °C for 2 hours. A peristaltic pump was used to maintain a consistent flow rate of 0.2 mL min^−1^, ensuring uniform silanization across the bead surfaces. This treatment facilitated the covalent grafting of amino-functional groups onto the silica matrix, effectively preparing the surface for enzyme immobilization. After silanization, the column was thoroughly flushed with methanol to remove unreacted APTES, followed by a final rinse with deionized water to eliminate residual solvents and prevent premature hydrolysis during subsequent enzyme loading steps.^25,26^ To confirm successful grafting of APTES, FTIR spectroscopy was performed on the functionalized beads. Characteristic absorption peaks corresponding to –NH_2_ bending (∼1560 cm^−1^) and Si–O–Si stretching (∼1000–1100 cm^−1^) were used as key indicators of successful aminosilane attachment. The appearance of these peaks, along with a reduction in surface hydrophilicity, confirmed the presence of the silane layer and supported effective surface modification. It is important to note that the concentration of methanol used during the washing step can significantly affect the stability of the grafted silane layer. Excessively concentrated methanol can lead to partial or complete desorption of loosely bound APTES molecules. Therefore, a diluted methanol solution (typically ≤50% v/v in water) should be used to effectively remove unreacted silane without compromising the integrity of the functionalized surface (Fig. 3).

##### 2.1.1.4 Covalent immobilization of laccase on functionalized glass beads

Laccase enzyme (sourced from *Trametes versicolor*) was immobilized onto the APTES-functionalized glass beads by circulating a buffered enzyme solution (1 mg mL^−1^ in 50 mM phosphate buffer, pH 5.0) through the packed column under mild agitation at 25 °C for 12 hours. The immobilization process was driven by a combination of electrostatic interactions and covalent bonding between the amino-functionalized silica surface and reactive residues on the laccase enzyme. Following the incubation period, unbound enzyme molecules were thoroughly removed by rinsing the beads with fresh phosphate buffer. The success of surface modification and enzyme immobilization was confirmed *via* Fourier-transform infrared (FTIR) spectroscopy, which revealed characteristic functional group signatures, and by protein activity assays, indicating retained enzymatic functionality on the immobilized surface.^27^

#### 2.1.2 Environmentally benign oxidation of HMF to FDCA *via* Co–Mn/AC in batch mode

The oxidation reaction was carried out in a 100 mL high-pressure stainless steel autoclave reactor (Parr Instrument Company) equipped with an overhead mechanical stirrer (500 rpm) and a pressure gauge to ensure uniform mixing and precise control of reaction conditions. A reaction suspension was prepared by dispersing 0.1 g of Co–Mn/AC-1 catalyst in 70 mL of an aqueous 1 mM 5-HMF solution. Atmospheric air was employed as the oxidant, and the reaction mixture was maintained under continuous stirring at 500 rpm for 5 hours. Upon completion, the solid catalyst was recovered by centrifugation, and the resulting supernatant was subjected to quantitative analysis using high-performance liquid chromatography (HPLC).^28,29^ To determine conversion efficiency and product distribution. The mixture was analyzed using an HPLC system (Jasco UV-4075) equipped with a C-18 column (4.6 × 250 mm) and a UV-Vis detector set at 265 nm. The mobile phase consisted of acetonitrile and 0.05 M sodium phosphate buffer in a 20 : 80 volumetric ratio, with the elution performed at a flow rate of 1 mL min^−1^ at 35 °C.

#### 2.1.3 Selective batch transformation of 5-HMF to FDCA *via* MnO_2_-functionalized resin

A reaction mixture was prepared by dispersing 0.1 g of MnO_2_–resin catalyst in 70 mL of an aqueous 1 mM 5-HMF solution. The oxidation was conducted under ambient air as the oxidant, with continuous stirring at 500 rpm for 12 hours to facilitate the catalytic conversion.^30^ Upon completion, the heterogeneous catalyst was separated *via* centrifugation, and the clear supernatant was collected for product analysis using high-performance liquid chromatography (HPLC) to evaluate conversion efficiency and product yield.^31,32^

The mixture was analyzed using an HPLC system (Jasco UV-4075) equipped with a C-18 column (4.6 × 250 mm) and a UV-Vis detector set at 265 nm. The mobile phase consisted of acetonitrile and 0.05 M sodium phosphate buffer in a 20 : 80 volumetric ratio, with the elution performed at a flow rate of 1 mL min^−1^ at 35 °C.

#### 2.1.4 Green conversion of 5-HMF to 2,5-FDCA using immobilized oxidative enzymes

A batch biocatalytic reaction was performed to assess the conversion efficiency of 5-hydroxymethylfurfural (5-HMF) to 2,5-furandicarboxylic acid (FDCA) using laccase in the presence of a redox mediator(McKenna *et al.*, 2017).^24^ The reaction mixture was prepared by dissolving 5 mM 5-HMF and 5 mM TEMPO in 20 mL of sodium acetate buffer (50 mM, pH 5.0). Sodium acetate buffer was used to maintain a stable acidic pH (pH 5.0), which is essential for optimal laccase activity, while TEMPO (2,2,6,6-tetramethylpiperidine-1-oxyl) served as a redox mediator to enhance the oxidation of 5-HMF by facilitating efficient electron transfer between the enzyme and the substrate. Laccase enzyme (20 mg) was introduced into the mixture, and the reactions were conducted at three different temperatures (30 °C, 40 °C, and 50 °C) to investigate the influence of thermal conditions on catalytic performance.^33^ Continuous stirring at 200 rpm was maintained to ensure homogeneous mixing, and ambient air was supplied at a flow rate of 5 mL min^−1^ to provide a constant source of molecular oxygen required for the oxidative transformation. Reaction samples were withdrawn at hourly intervals, and conversion progress was monitored by high-performance liquid chromatography (HPLC), enabling quantification of intermediate and final product formation, including FDCA.^34^ The analysis was performed using an HPLC system (Jasco UV-4075) equipped with a C-18 column (4.6 × 250 mm) and a UV-Vis detector set at 265 nm, with a mobile phase of acetonitrile and 0.05 M sodium phosphate buffer (20 : 80, v/v), eluted at 1 mL min^−1^ at 35 °C.

#### 2.1.5 Sustained enzymatic oxidation of 5-HMF in a packed-bed reactor with immobilized laccase

Laccase enzyme immobilized on glass bead supports was employed for the continuous biocatalytic oxidation of 5-hydroxymethylfurfural (5-HMF) to 2,5-furandicarboxylic acid (FDCA). The feed solution consisted of 5 mM 5-HMF and 5 mM TEMPO (2,2,6,6-tetramethylpiperidinyloxy) dissolved in 20 mL of sodium acetate buffer (50 mM, pH 5.0). This reaction mixture was introduced into a glass column (20 cm length, 1 cm inner diameter) packed with 15 g of 1 mm diameter laccase-functionalized glass beads, using a peristaltic pump at a controlled flow rate of 0.02 mL min^−1^ (Balboa *et al.*, 2022). A continuous aerobic environment was maintained by supplying air at 5 mL min^−1^, essential for sustaining the oxidative activity of laccase. The effluent product stream was collected from the outlet of the column, and reaction progress was monitored by high-performance liquid chromatography (HPLC) to quantify the formation of FDCA and assess the catalytic performance of the immobilized enzyme system.^35,36^

#### 2.1.6 Quantitative determination of FDCA *via* high-performance liquid chromatography (HPLC)

The analysis of 2,5-furandicarboxylic acid (FDCA) was performed using High-Performance Liquid Chromatography (HPLC) equipped with a UV-Vis detector set at 265 nm.^37^ A C18 reverse-phase column (4.6 × 250 mm, 5 μm particle size) was utilized, providing effective separation due to hydrophobic interactions with the analyte. The mobile phase consisted of acetonitrile and 0.05 M sodium phosphate buffer (pH adjusted to ∼3.0) in a 20 : 80 (v/v) ratio, maintaining the acidic environment necessary for FDCA stability and optimal peak resolution.^38^

Quantification was achieved using external standard calibration with FDCA standards, allowing for the accurate determination of FDCA concentrations in the samples. Prior to analysis, samples were filtered through a 0.45 μm membrane filter to remove particulates. System suitability tests were conducted to verify that parameters such as retention time, theoretical plates, and peak symmetry met acceptable criteria. The method was validated for linearity, accuracy, precision, limit of detection (LOD), limit of quantification (LOQ), and robustness to ensure reliability and reproducibility.^39,40^

## 3 Result and discussion

This study presents a sustainable and integrated biocatalytic–chemical approach for the conversion of 5-hydroxymethylfurfural (5-HMF) to 2,5-furandicarboxylic acid (FDCA), fully aligned with the principles of green chemistry and circular biorefinery. Leveraging biomass-derived 5-HMF as a renewable feedstock, the process operates under mild aqueous-phase conditions and utilizes recyclable heterogeneous catalysts (*e.g.*, Co–Mn/activated carbon and MnO_2_) to facilitate the stepwise oxidation of HMF to FDCA *via* key intermediates such as 5-hydroxymethyl-2-furancarboxylic acid (HMFCA) and 5-formyl-2-furancarboxylic acid (FFCA).

The use of molecular oxygen as a benign oxidant, coupled with low-temperature sealed batch reactor operations, enhances product selectivity while reducing energy demands and greenhouse gas emissions. Importantly, the heterogeneous catalysts demonstrated excellent reusability over multiple cycles, indicating a robust and economically favorable oxidation platform.

Furthermore, the integration of enzymatic and catalytic oxidation steps within a single workflow underscores the process's versatility and scalability. This hybrid methodology not only minimizes environmental impact but also supports the sustainable synthesis of FDCA, a key monomer for bio-based polyesters such as polyethylene furanoate (PEF), a green alternative to fossil-derived PET. The findings underscore the feasibility of this dual-function system as a practical route for renewable polymer precursors and contribute meaningfully to the advancement of green and circular chemical manufacturing:

### 3.1 FTIR characterization of silane-treated glass beads for enzyme immobilization

**Fig. 4:**
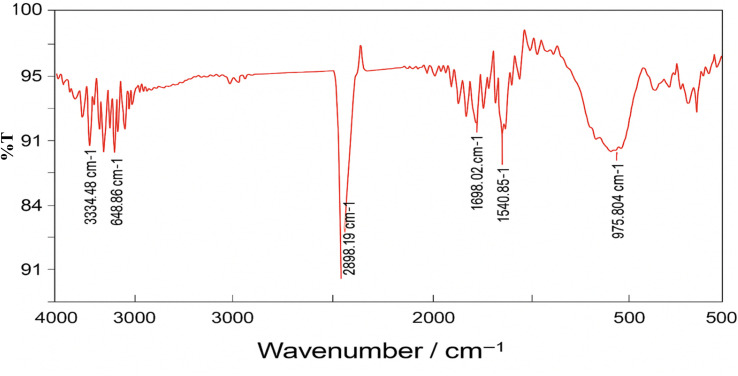
FTIR spectrum of glass beads functionalized with APTES (3-aminopropyltriethoxysilane).

The successful surface functionalization of glass beads with 3-aminopropyltriethoxysilane (APTES) was confirmed through Fourier-transform infrared (FTIR) spectroscopy, as shown in Table 1 and Fig. 4. The FTIR spectrum exhibited several characteristic peaks corresponding to functional groups introduced *via* the silanization process.

**Table 1:** Comparison of reported catalytic methods for 5-HMF to FDCA conversion

System	Catalyst type	Mode	Conditions	Yield/selectivity	Limitations
Noble metal-based catalysts (*e.g.*, Pt/C, Ru/C)	Heterogeneous (metal)	Batch	120–160 °C, 10–30 bar O_2_	>90% yield	Expensive, limited scalability, harsh conditions
TEMPO/NaOCl system	Homogeneous (chemical)	Batch	Ambient T, pH 9–11	Moderate	Toxic reagents, waste disposal issues
Biocatalysis with free laccase	Enzymatic	Batch	Mild (30–40 °C, pH 5)	Low–moderate	Slow kinetics, enzyme instability, limited reuse
Immobilized laccase in PBR	Enzymatic	Continuous	Mild (30–40 °C, pH 5)	Moderate	Improved stability, but limited by enzyme-only redox potential
This study: Co–Mn/AC + immobilized laccase	Hybrid (inorganic + enzyme)	Batch + continuous	Mild–moderate (40–60 °C), atmospheric pressure	High (up to ∼90%) with enhanced selectivity	Reduces steps, cost-effective, scalable, low energy input

**Fig. 5:**
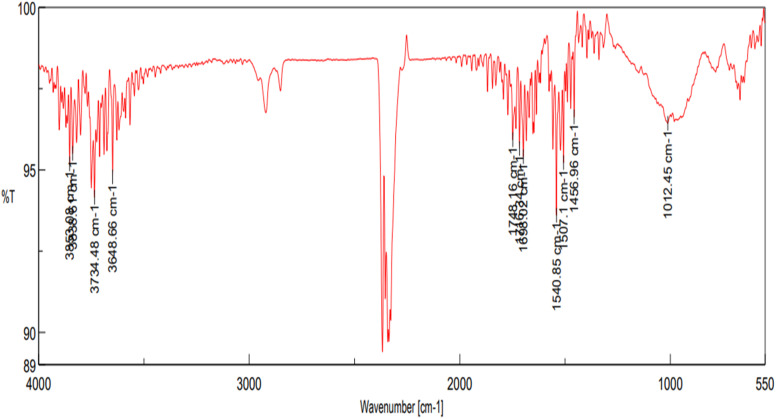
FTIR spectrum of untreated glass beads (without APTES functionalization).

A broad absorption band observed in the range of 3353.86 cm^−1^ to 3648.66 cm^−1^ corresponds to O–H and N–H stretching vibrations, indicating the presence of surface hydroxyl groups and primary amines from the grafted APTES molecules.^41^ The strong and sharp peak at 2925.57 cm^−1^ is attributed to the asymmetric stretching vibrations of –CH_2_ groups in the propyl chain of APTES, confirming the incorporation of alkyl linkers onto the surface.^41^ The peak at 1698.92 cm^−1^ can be assigned to C=O stretching, which may arise from minor surface oxidation or residual amine–carbonyl interactions. The band near 1540.85 cm^−1^ is characteristic of N–H bending vibrations, further supporting the presence of primary amine groups. Additionally, a distinct band at 976.804 cm^−1^ corresponds to Si–O–Si symmetric stretching, confirming the formation of siloxane linkages between the silane and the silica surface^42^ (as shown in Fig. 4 and 5). Collectively, these spectral features confirm the effective covalent attachment of APTES to the activated glass bead surface, providing amine-functional sites suitable for subsequent enzyme immobilization (Tables 2 and 3).

**Table 2:** Comparison of Co–Mn/AC catalyst activity with literature-reported noble metal catalysts for FDCA synthesis

Catalyst system	FDCA yield (%)	Temperature (°C)	O_2_ pressure (bar)	Key features
Pt/C (literature)	85–95	120–160	10–30	Noble metal; high *T* & pressure required
Au/CeO_2_ (literature)	85–95	120–160	10–30	Noble metal; high *T* & pressure required
Co–Mn/AC + laccase (this work)	88–90	40–60	1 (atm)	Non-noble metal; milder, eco-friendly

**Table 3:** Comparison of the present study with existing literature-reported processes

Parameter	Literature-reported process	Present manuscript process
Mode of operation	Primarily batch mode, often with limited scalability	Includes both batch and continuous modes, tested under various controlled conditions
Reactor type	Conventional glass reactors or flasks	An autoclave reactor is used for reactions under controlled temperature and pressure
Reaction conditions	Often at higher temperatures and pressures, uncontrolled aeration	Optimized temperatures, controlled aeration, and ambient pressure
Catalytic system	Enzymes or metal catalysts used without mediators or under non-ideal pH conditions	Laccase + TEMPO system with pH-optimized sodium acetate buffer for enhanced biocatalytic oxidation
Product monitoring	Limited analytical detail or end-point analysis only	Time-resolved sampling with HPLC quantification using Jasco UV-4075, C-18 column, UV at 265 nm
Scalability and process control	Not easily scalable due to batch limitations	Continuous flow-compatible and scalable setup with stirred batch and pressurized reactors
Environmental impact	May involve harsher chemicals or conditions	Ambient air is used as an oxidant, a mild buffer system, and a recyclable catalyst

### 3.2 Study of the effect of physical parameters of enzymatic reaction: batch conversion of 5-HMF to FDCA

#### 3.2.1 Temperature-dependent performance of the catalytic/biocatalytic system

The enzymatic conversion of 5-HMF to FDCA is highly temperature-dependent, as temperature influences the activity and stability of laccase. At the same time, it is true for all enzymatic reactions that temperature impacts reaction rates (as per the Arrhenius equation); the optimal temperature range for this specific conversion needs to be carefully determined. To investigate this, the enzymatic oxidation was carried out at temperatures ranging from 30 to 50 °C, while maintaining fixed conditions of 1 mg mL^−1^ enzyme concentration, pH 5, and a reaction duration of 12 hours (Cajnko *et al.*, 2020). This temperature range was chosen to capture both the potential increase in enzyme activity and the point at which enzyme denaturation could begin, providing insights into the most efficient operating conditions for maximizing FDCA yield.^43^

**Fig. 6:**
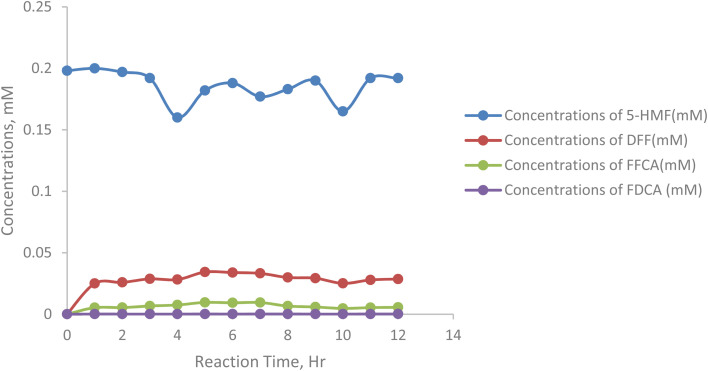
Concentration of 5-HMF oxidation product at 30 °C in batch reaction.

Fig. 6 shows the concentration changes of four compounds, 5-HMF, DFF, FFCA, and FDCA, during a 12-hour reaction at 30 °C under controlled conditions. Initially, the concentration of 5-HMF increases slightly but then decreases steadily over time, from 0.198 mM at hour 0 to a minimum of 0.165 mM at hour 10, indicating its transformation into other products. The concentration of DFF rises steadily from zero to a peak of 0.0343 mM at hour 5, then fluctuates around 0.03 mM. FFCA also increases progressively, reaching a peak of 0.0096 mM at hour 5 before stabilizing. In contrast, the concentration of FDCA remains low throughout, starting at 0.000145 mM and only reaching 0.000173 mM by hour 12. This suggests that DFF and FFCA are the primary products, while FDCA is a minor product formed at a slower rate. Overall, the data reflect the progression of 5-HMF conversion into DFF and FFCA, with FDCA forming at a significantly lower rate (Tables 4 and 5).

**Table 4:** Summary of product concentration (mM) at 30 °C temperature

Sample/h	Concentration of 5-HMF (mM)	Concentration of DFF (mM)	Concentration of FFCA (mM)	Concentration of FDCA (mM)
0	0.198	0	0	0
1	0.2	0.025	0.00531	0.000145
2	0.197	0.0259	0.00539	0.000124
3	0.192	0.0287	0.0067	0.000111
4	0.16	0.0282	0.00753	0.000134
5	0.182	0.0343	0.0096	0.000154
6	0.188	0.0339	0.00927	0.000139
7	0.177	0.0332	0.00949	0.00016
8	0.183	0.0299	0.00664	0.00014
9	0.19	0.0293	0.00588	0.000133
10	0.165	0.0251	0.00471	0.000139
11	0.192	0.0279	0.00538	0.000164
12	0.192	0.0286	0.00558	0.000173

**Table 5:** Summary of product concentration (mM) at 40 °C temperature

Sample/h	Concentration of 5-HMF (mM)	Concentrations of DFF (mM)	Concentrations of FFCA (mM)	Concentrations of FDCA (mM)
0	0.245	0	0	0
1	0.211	0.0216	0.00256	0
2	0.202	0.0265	0.00467	0.0000456
3	0.223	0.0274	0.00414	0.0000348
4	0.237	0.0337	0.00581	0.0000577
5	0.219	0.0316	0.00547	0.0000557
6	0.245	0.0373	0.00648	0.0000536
7	0.238	0.0371	0.0079	0.0000352
8	0.247	0.043	0.00795	0.0000428
9	0.232	0.0424	0.0091	0.0000644
10	0.235	0.0457	0.00973	0.0000644
11	0.236	0.0466	0.0103	0.0000408
12	0.226461	0.00527	0.0103	0.000079

**Fig. 7:**
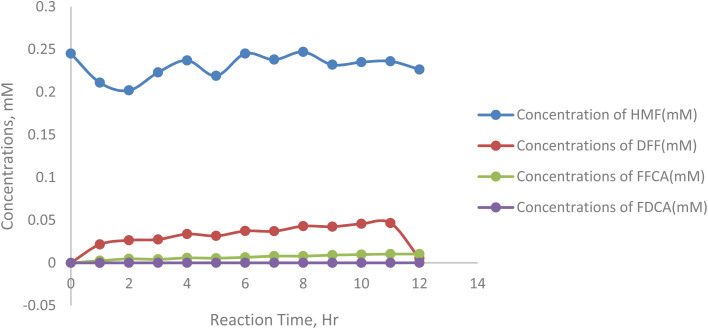
Concentration dynamics of 5-HMF oxidation products at 40 °C.

Fig. 7 represents the concentration of 5-HMF, the primary substrate, which decreases steadily from 0.198 mM at hour 0 to 0.192 mM at hour 12, with minor fluctuations at specific points (*e.g.*, an increase from 0.182 mM at hour 5 to 0.188 mM at hour 6), suggesting kinetic inconsistencies or substrate inhibition. DFF, the first oxidation intermediate, accumulates rapidly, peaking at 0.0343 mM at hour 5, reflecting efficient oxidation of 5-HMF but a slower conversion to FFCA. FFCA forms more gradually, increasing to 0.0096 mM at hour 5 and fluctuating afterward, with a slight decline after hour 7, indicating a barrier in the pathway from DFF to FFCA. The final product, FDCA, accumulates minimally, reaching only 0.000173 mM at hour 12, highlighting significant challenges in the final oxidation step.

At an initial substrate concentration of 250 mM, the enzymatic oxidation process exhibits significant variations in product formation with changing temperatures. At lower temperatures, the consumption of 5-HMF is slower, resulting in higher residual concentrations and reduced product formation. As the temperature increases, the oxidation of 5-HMF to DFF accelerates, with DFF accumulation peaking at 0.0527 mM at 12 hours. FFCA formation is also temperature-dependent, with higher temperatures favouring its production, reaching a maximum of 0.0103 mM. However, the conversion of FFCA to FDCA remains limited across all temperatures, with FDCA showing minimal accumulation, peaking at 7.9 × 10^−5^ mM at 12 hours. These observations suggest that while increased temperatures enhance intermediate product formation, the final oxidation step to FDCA is less.

**Fig. 8:**
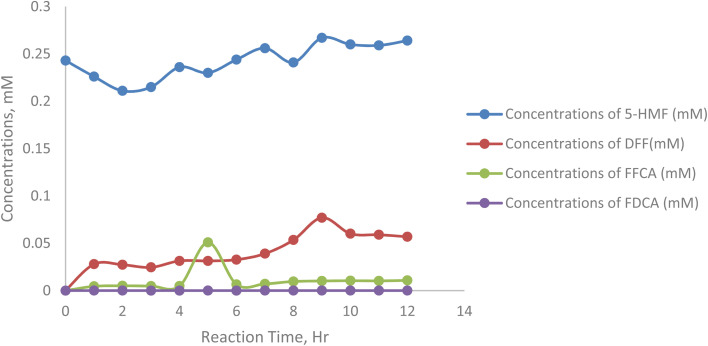
Concentration dynamics of oxidation intermediates and FDCA at 50 °C.

Fig. 8 shows that at 50 °C, the enzymatic oxidation process exhibits distinct variations in product concentrations, reflecting temperature-dependent reaction kinetics. The concentration of 5-HMF fluctuates over time because there is water loss by evaporation from the reaction mixture, starting at 0.243 mM, peaking at 0.267 mM at hour 9, and ending at 0.264 mM at hour 12, suggesting incomplete substrate consumption due to potential substrate inhibition or enzyme instability at higher temperatures. DFF, the first oxidation intermediate, shows steady accumulation, reaching a maximum of 0.077 mM at hour 9, indicating efficient initial oxidation of 5-HMF but slower progression to FFCA. FFCA formation is gradual, peaking at 0.0108 mM at hour 12, highlighting a secondary barrier in its conversion to FDCA. The final product, FDCA, accumulates minimally, with its concentration increasing slightly to 0.0000677 mM by hour 12, underscoring significant challenges in achieving complete oxidation at elevated temperatures (Table 6).

**Table 6:** Summary of product concentration (mM) at 50 °C temperature

Sample/h	Concentrations of 5-HMF (mM)	Concentrations of DFF (mM)	Concentrations of FFCA (mM)	Concentrations of FDCA (mM)
0	0.243	0	0	0
1	0.226	0.028	0.00456	0.0000472
2	0.211	0.0273	0.00509	0.0000384
3	0.215	0.0246	0.00484	0.0000302
4	0.236	0.0314	0.00486	0.0000259
5	0.23	0.0314	0.051	0.0000348
6	0.244	0.0327	0.00654	0.0000165
7	0.256	0.0391	0.00711	0.0000413
8	0.241	0.0535	0.00965	0.000062
9	0.267	0.077	0.0102	0.0000717
10	0.26	0.0602	0.0105	0.0000723
11	0.259	0.059	0.0103	0.0000668
12	0.264	0.0569	0.0108	0.0000677

**Fig. 9:**
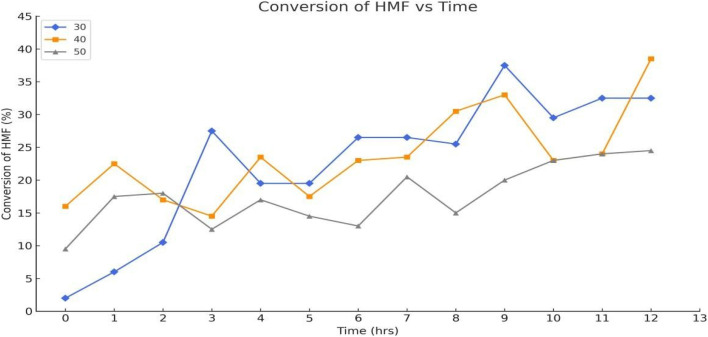
Conversion of 5-hydroxymethylfurfural at different temperatures.

Fig. 9 represents the conversion of 5-hydroxymethylfurfural (5-HMF) under different temperature conditions (30 °C, 40 °C, and 50 °C) in the presence of TEMPO and laccase. As the temperature increased, the conversion rate of 5-HMF also showed a marked improvement, which can be attributed to the enhanced activity of laccase at higher temperatures. At 30 °C, the conversion was relatively slow, but at 50 °C, the conversion reached its peak, suggesting that the enzyme activity is significantly influenced by temperature. However, it is important to consider that excessively high temperatures might also lead to enzyme denaturation, limiting the maximum conversion. These results indicate that optimizing temperature is crucial for maximizing the efficiency of the conversion process.

**Fig. 10:**
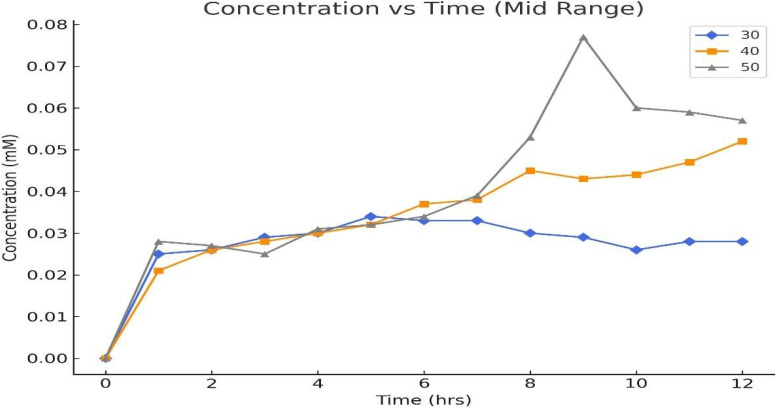
Concentrations of 2,5-diformylfuran at different temperatures.

Fig. 10 shows the concentration of 2,5-diformylfuran (DFF) at different temperatures (30 °C, 40 °C, and 50 °C), reflecting the early-stage oxidation of 5-HMF in the enzymatic pathway. DFF formation is a crucial intermediate step, and its accumulation can indicate a first step in further oxidation to FFCA and FDCA. At 30 °C, the low concentrations of DFF suggest reduced enzymatic activity at suboptimal temperatures. At 40 °C, a higher concentration of DFF is observed, likely due to optimal laccase activity facilitating the conversion of 5-HMF to DFF. However, at 50 °C, a decline in DFF levels is apparent, possibly due to enzyme denaturation or reduced mediator (TEMPO) efficiency at elevated temperatures. The limited conversion of DFF into downstream products suggests challenges in achieving complete oxidation under the tested conditions.

**Fig. 11:**
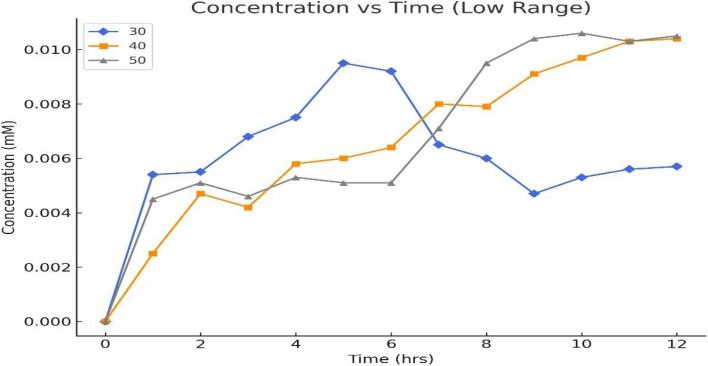
Concentrations of formyl furan carboxylic acid at the different temperatures.

The results in Fig. 11, depicting the concentrations of Formyl Furan carboxylic Acid (FFCA) at different temperatures (30 °C, 40 °C, and 50 °C), provide insights into the intermediate stages of 5-HMF oxidation. FFCA formation is an expected step in the enzymatic pathway from 5-HMF to FDCA, catalyzed by laccase. At 30 °C, the reaction likely proceeds slowly due to reduced enzymatic activity at suboptimal temperatures. At 40 °C, the observed higher concentrations of FFCA suggest an optimal balance of enzymatic activity and substrate conversion. However, at 50 °C, the reduced FFCA levels indicate potential thermal denaturation of the enzyme or a shift in equilibrium, limiting intermediate formation. Additionally, the accumulation of FFCA without significant FDCA formation (as seen in Fig. 11) suggests incomplete conversion along the oxidation pathway. These results highlight the need for process optimization, particularly temperature control, to ensure efficient conversion of intermediates into the final product, FDCA.

**Fig. 12:**
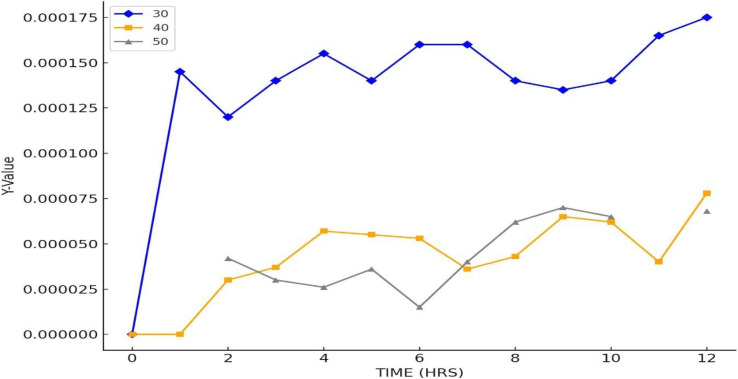
Concentrations of 2,5-furan dicarboxylic acid at different temperatures.

The results in Fig. 12 show minimal FDCA formation at all tested temperatures (30 °C, 40 °C, and 50 °C), indicating suboptimal reaction conditions for enzymatic conversion. At 30 °C, the enzyme activity is likely insufficient, while at 50 °C, thermal denaturation of laccase may reduce its effectiveness. The observed nanomolar concentrations suggest extremely low reaction efficiency, potentially due to insufficient enzyme–substrate interaction, high substrate concentration causing inhibition, or mediator (TEMPO) degradation. The peak conversion at 40 °C aligns with typical laccase activity, but overall yields are still negligible. Improvements such as optimizing substrate concentration, lowering substrate levels, extending reaction times, and stabilizing TEMPO are necessary to enhance FDCA production under these conditions.

#### 3.2.2 Effect of substrate concentration on product formation and catalytic efficiency

The impact of substrate concentration on the conversion of 5-HMF to FDCA was investigated by altering 5-HMF concentrations (25, 75, 125, 175, 250 mmole 5-HMF) under controlled enzymatic conditions.

At lower 5-HMF concentrations (25 mM & 250 mM), lower conversion rates but higher FDCA yields were observed compared to those at higher 5-HMF concentrations (125 mM, 175 mM, & 250 mM). Specifically, at 125 mM 5-HMF, the conversion efficiency reached 98% with an FDCA yield (calculated as 13 molar% of initial 5-HMF converted to FDCA) of 11.3 mol (11.3 mol FDCA formed per 100 mol of 5-HMF added), whereas at 250 mM, the yield of product decreased to 0.01% despite substantial substrate consumption. This suggests that while higher substrate concentrations theoretically offer more reactants for the enzymatic process, there may be inhibition by high substrate concentration or saturation effects at play, leading to reduced overall efficiency.^44,45^

The decrease in yield at higher concentrations can be attributed to several factors, where excess 5-HMF might interfere with the enzymatic activity or saturation effects, where the enzyme becomes fully occupied with 5-HMF molecules. Moreover, the reaction conditions, such as catalyst amount and buffer capacity (pH 5), become critical at higher substrate concentrations, affecting the overall conversion efficiency.^46^

**Fig. 13:**
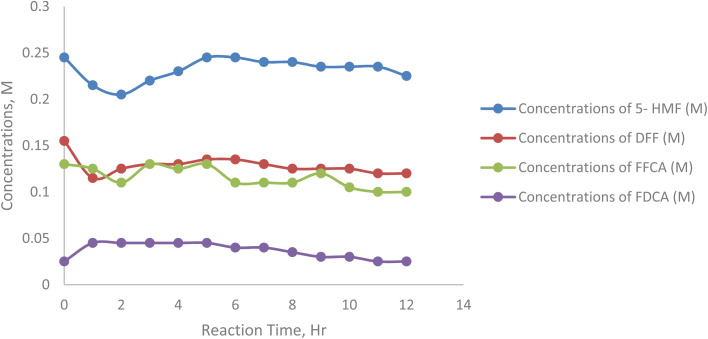
Concentrations of 5-hydroxymethylfurfural at 25, 75, 125, 175, 250 mM substrate concentrations.

Fig. 13 shows initial concentration of 5-HMF strongly influences the reaction kinetics. At lower substrate concentrations (*e.g.*, 125 mM), the enzyme efficiently converted 5-HMF into intermediates and FDCA, resulting in faster depletion of 5-HMF over time. As the substrate concentration increased (*e.g.*, 0.75 mM), the conversion rate slowed down, indicating substrate inhibition or saturation of the enzyme active sites. Higher initial concentrations of 5-HMF (*e.g.*, 75 M and 225 M) showed a plateau in 5-HMF depletion, suggesting a limitation in enzymatic activity due to substrate inhibition. Excess 5-HMF may hinder enzyme activity by reducing the availability of free active sites (Table 7).

**Table 7:** Concentrations of 5-hydroxymethyl furfural (HMF) 25, 75, 125, 175, 250 mM substrate concentrations

Sample/h	Concentrations of 5-HMF (M)	Concentrations of DFF (M)	Concentrations of FFCA (M)	Concentrations of FDCA (M)
0	0.245	0.155	0.13	0.025
1	0.215	0.115	0.125	0.045
2	0.205	0.125	0.11	0.045
3	0.22	0.13	0.13	0.045
4	0.23	0.13	0.125	0.045
5	0.245	0.135	0.13	0.045
6	0.245	0.135	0.11	0.04
7	0.24	0.13	0.11	0.04
8	0.24	0.125	0.11	0.035
9	0.235	0.125	0.12	0.03
10	0.235	0.125	0.105	0.03
11	0.235	0.12	0.1	0.025
12	0.225	0.12	0.1	0.025

The results underline the critical role of initial substrate concentration in the enzymatic oxidation of 5-HMF. Lower concentrations favor efficient conversion, while higher concentrations lead to substrate inhibition. These findings provide essential insights into optimizing substrate levels to maximize FDCA yield and reaction efficiency in enzymatic processes.

**Fig. 14:**
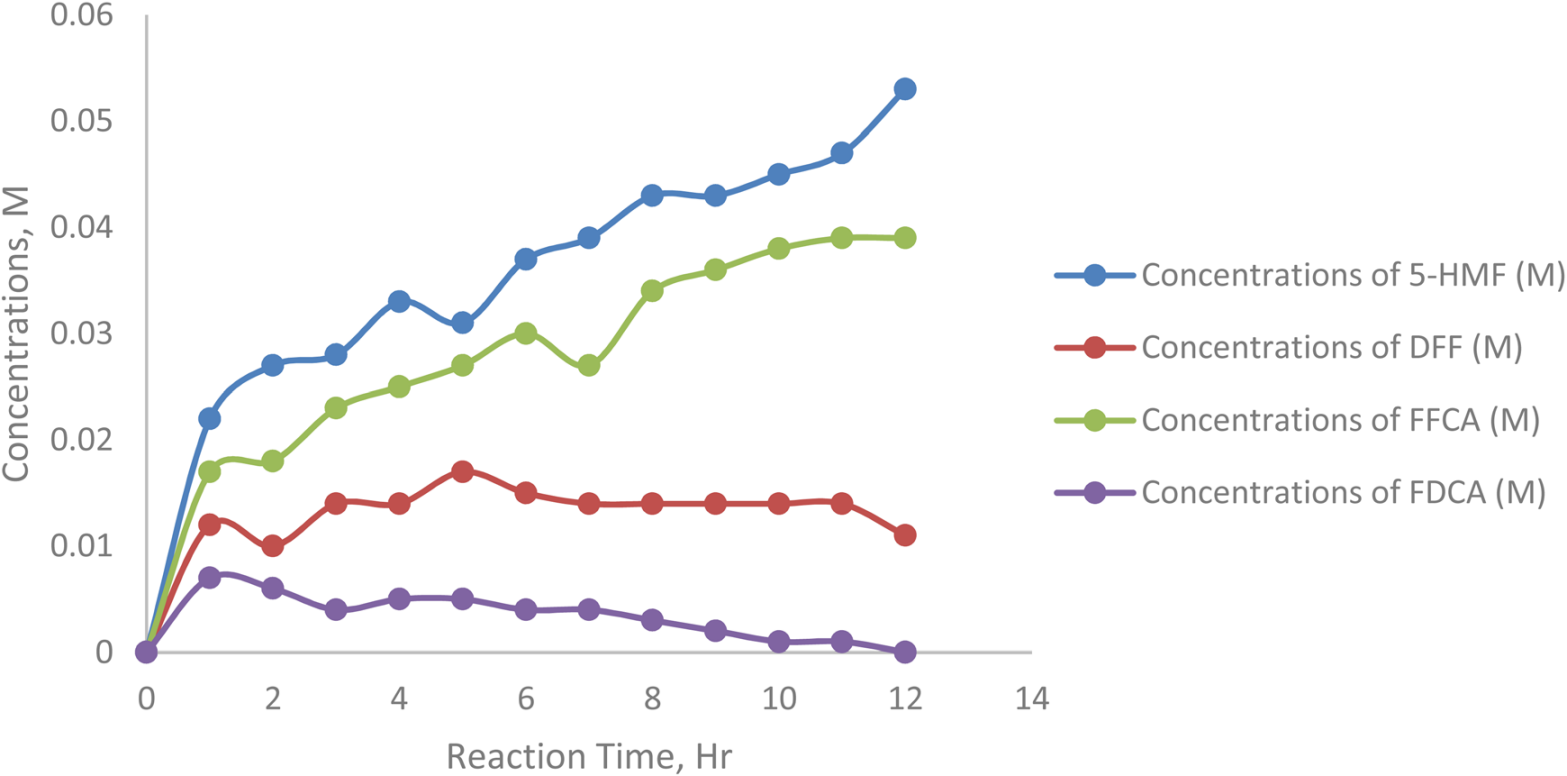
Concentrations of 2,5-diformyl furan at 25, 75, 125, 175, 250 mM substrate concentrations.

Fig. 14 represents the effect of varying initial substrate concentrations (25 mM, 75 mM, 125 mM, 175 mM, and 225 mM 5-HMF) on the formation of 2,5-diformylfuran (DFF) during enzymatic oxidation. The experiments were conducted under fixed conditions with 5 mM TEMPO as a mediator, 20 mL sodium acetate buffer (50 mM, pH 5), air flow at 5 mL min^−1^, 20 mg laccase, 40 °C, and 200 rpm stirring (Table 8).

**Table 8:** Concentrations of 2,5-diformylfuran at 25, 75, 125, 175.250 mM substrate concentrations

Sample/h	Concentrations of 5-HMF (M)	Concentrations of DFF (M)	Concentrations of FFCA (M)	Concentrations of FDCA (M)
0	0.000	0.000	0.000	0.000
1	0.022	0.012	0.017	0.007
2	0.027	0.010	0.018	0.006
3	0.028	0.014	0.023	0.004
4	0.033	0.014	0.025	0.005
5	0.031	0.017	0.027	0.005
6	0.037	0.015	0.030	0.004
7	0.039	0.014	0.027	0.004
8	0.043	0.014	0.034	0.003
9	0.043	0.014	0.036	0.002
10	0.045	0.014	0.038	0.001
11	0.047	0.014	0.039	0.001
12	0.053	0.011	0.039	0.000

At lower substrate concentrations (*e.g.*, 125 mM and 175 mM), the DFF concentration increased rapidly in the early stages of the reaction and then plateaued. This suggests efficient oxidation of 5-HMF to DFF, which subsequently progresses to further oxidation products such as FFCA and FDCA. However, at higher substrate concentrations (*e.g.*, 75 mM), the accumulation of DFF was more pronounced, indicating a barrier to further oxidation. This is likely due to substrate inhibition, where excess 5-HMF competes with DFF for enzyme active sites or reduces the effectiveness of the mediator (TEMPO).

Excessively high substrate levels not only lead to incomplete conversion but also reduce the efficiency of intermediate utilization. These findings underscore the critical role of substrate concentration in modulating the reaction pathway and maximizing the yield of FDCA.

**Fig. 15:**
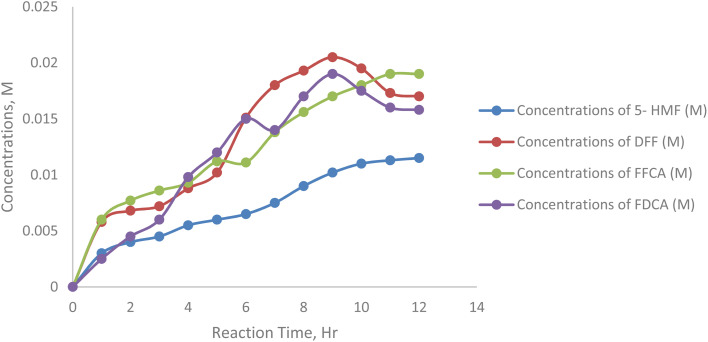
Concentrations of formyl furan carboxylic acid at 25, 75, 125, 175, 250 mM substrate concentration.

Fig. 15 shows the effect of varying initial 5-HMF concentrations (25–250 mM) on FFCA accumulation during enzymatic oxidation. FFCA peaked at moderate 5-HMF levels (125 and 175 mM), indicating efficient DFF oxidation but limited conversion to FDCA. Minimal FFCA at 25 mM suggests rapid full oxidation, while reduced levels at 75 mM and 250 mM point to substrate inhibition or enzyme saturation. These results emphasize the importance of optimizing 5-HMF concentration to ensure efficient intermediate conversion and maximize FDCA yield (Table 9).

**Table 9:** Concentrations of formyl furan carboxylic acid at 25, 75, 125, 175, 250 mM substrate concentration

Sample/h	Concentrations of 5-HMF (M)	Concentrations of DFF (M)	Concentrations of FFCA (M)	Concentrations of FDCA (M)
0	0.000	0.000	0.000	0.000
1	0.003	0.0058	0.006	0.0025
2	0.004	0.0068	0.0077	0.0045
3	0.0045	0.0072	0.0086	0.006
4	0.0055	0.0088	0.0093	0.0098
5	0.006	0.0102	0.0112	0.012
6	0.0065	0.0151	0.0111	0.015
7	0.0075	0.018	0.0138	0.014
8	0.009	0.0193	0.0156	0.017
9	0.0102	0.0205	0.017	0.019
10	0.011	0.0195	0.018	0.0175
11	0.0113	0.0173	0.019	0.016
12	0.0115	0.017	0.019	0.0158

**Fig. 16:**
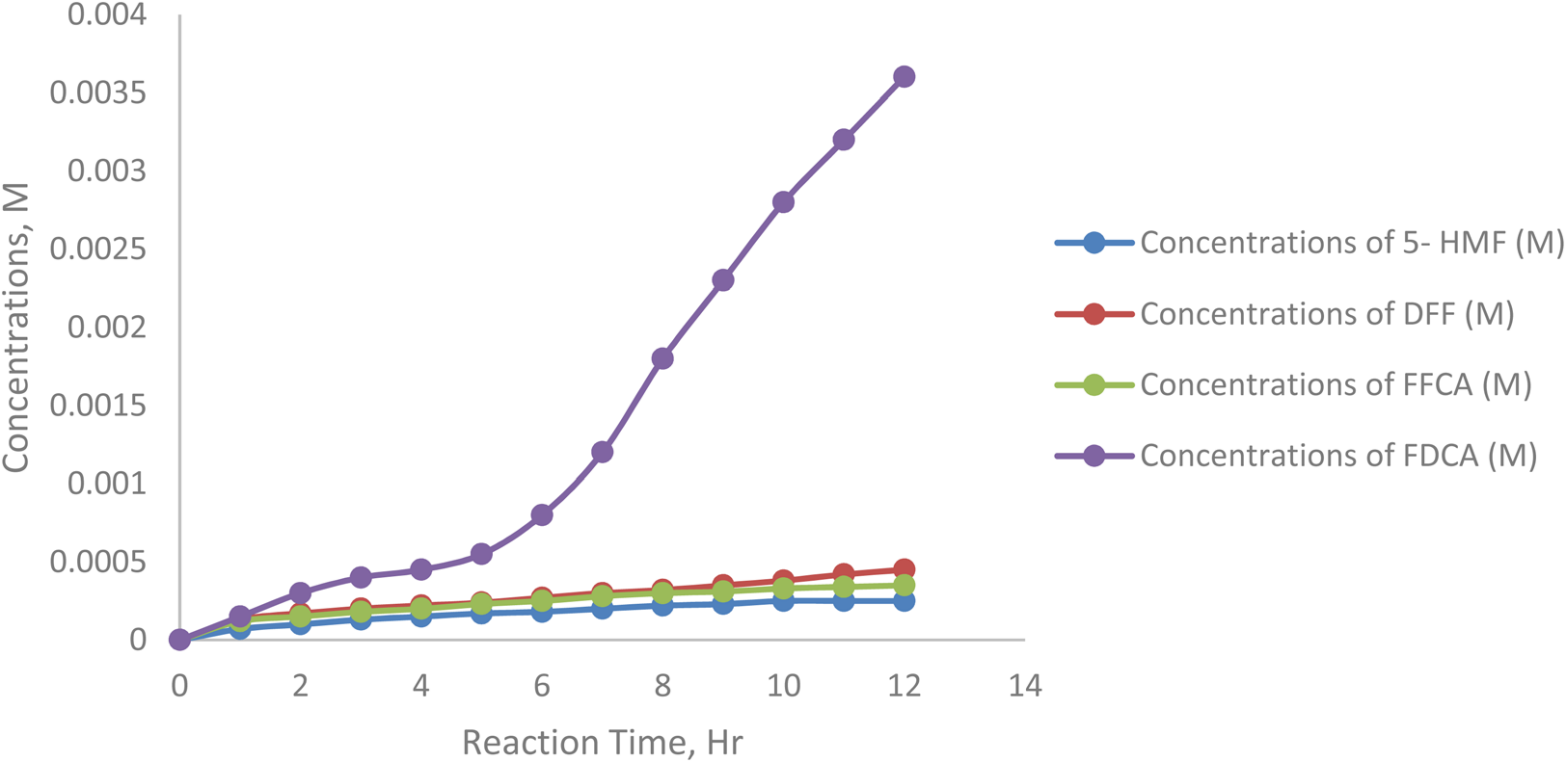
Concentrations of 2,5-furan dicarboxylic acid at 25, 75, 125, 175, 250 mM substrate concentration.

Fig. 16 represents the effect of varying initial 5-HMF 25, 75, 125, 175, 225 mM on the formation of 2,5-furandicarboxylic acid (FDCA) under enzymatic oxidation conditions. The reaction setup involved 5 mM TEMPO as a mediator, 20 mL sodium acetate buffer (50 mM, pH 5), 5 mL min^−1^ air flow, 20 mg laccase, at 40 °C and 200 rpm stirring.

At lower substrate concentrations (*e.g.*, 125 mM and 175 mM), FDCA formation was consistent and reached higher levels compared to higher substrate concentrations. This indicates efficient oxidation of 5-HMF through intermediates like DFF and FFCA to FDCA. The lower concentrations allowed the enzyme to function optimally, avoiding substrate inhibition and ensuring complete oxidation of intermediates. At higher substrate concentrations (*e.g.*, 75 M), FDCA accumulation was significantly reduced, likely due to substrate inhibition or enzyme active site saturation, which impeded the oxidation of intermediates to FDCA (Table 10).

**Table 10:** Concentrations of 2,5-furan dicarboxylic acid at 25, 75, 125, 175, 250 mM substrate concentrations

Sample/h	Concentrations of 5-HMF (M)	Concentrations of DFF (M)	Concentrations of FFCA (M)	Concentrations of FDCA (M)
0	0.00000	0.00000	0.00000	0.00000
1	0.00007	0.00013	0.00012	0.00015
2	0.00010	0.00017	0.00015	0.00030
3	0.00013	0.00020	0.00018	0.00040
4	0.00015	0.00022	0.00020	0.00045
5	0.00017	0.00024	0.00023	0.00055
6	0.00018	0.00027	0.00025	0.00080
7	0.00020	0.00030	0.00028	0.00120
8	0.00022	0.00032	0.00030	0.00180
9	0.00023	0.00035	0.00031	0.00230
10	0.00025	0.00038	0.00033	0.00280
11	0.00025	0.00042	0.00034	0.00320
12	0.00025	0.00045	0.00035	0.00360

The results emphasize that excessive 5-HMF levels can lead to incomplete oxidation and reduced FDCA yield. Careful control of substrate concentration is essential for maximizing FDCA yield and ensuring process efficiency in enzymatic oxidation reactions.

#### 3.2.3 Selective oxidation of laboratory-derived 5-HMF to FDCA under batch conditions

The study was conducted using in-house produced 0.1 mM 5-HMF and 1.0 mg mL^−1^ laccase concentration. The conversion of 5-HMF was investigated at 40 °C temperature and stirring at 200 rpm. The conversion of 5-HMF was 83.4 mol, and the FDCA yield was 1.5 mol in 12 hours. This concentration likely represents the lower end of the substrate range typically tested in such enzymatic conversion.^47^

The results of in-house-generated 5-HMF and pure 5-HMF differed. The chromatograms of both samples were recorded. It is apparent from the chromatogram that there is a presence of impurities in the in-house generated product compared to pure 5-HMF.

**Fig. 17:**
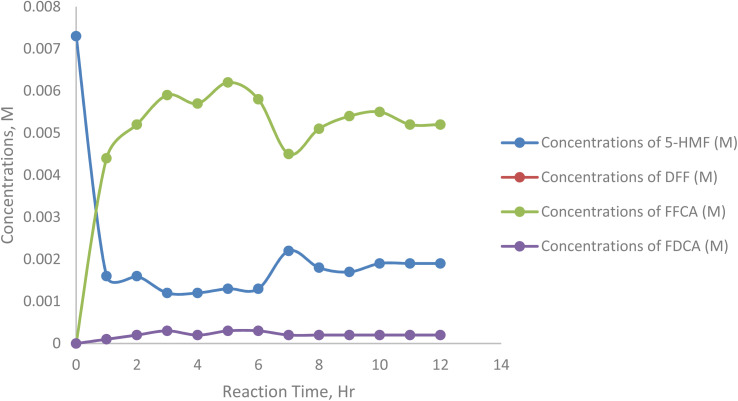
The concentration of FDCA, FFCA, DFF, and 5-HMF with respect to time.

Fig. 17 and Table 11 illustrate the time-resolved conversion profile of raw 5-HMF during enzymatic oxidation, showing the concentrations of FDCA, FFCA, DFF, and 5-HMF over 12 hours. Throughout the reaction, 5-HMF concentration remained high (∼80–88%), indicating minimal substrate consumption. FFCA yield peaked early (around 3–5 hours at ∼60 mol) and then declined, showing its role as a key intermediate. DFF levels were moderate (10–25%), peaking at 7 hours, while FDCA yield stayed very low (<5%) throughout the process. This suggests that the oxidation of FFCA to FDCA was inefficient, likely due to enzyme limitations or inhibition effects.

**Table 11:** The concentration of FDCA, FFCA, DFF, and 5-HMF with respect to time

Sample/h	Concentrations of 5-HMF (M)	Concentrations of DFF (M)	Concentrations of FFCA (M)	Concentrations of FDCA (M)
0	0.0073	0.0000	0.0000	0.0000
1	0.0016	0.0044	0.0044	0.0001
2	0.0016	0.0052	0.0052	0.0002
3	0.0012	0.0059	0.0059	0.0003
4	0.0012	0.0057	0.0057	0.0002
5	0.0013	0.0062	0.0062	0.0003
6	0.0013	0.0058	0.0058	0.0003
7	0.0022	0.0045	0.0045	0.0002
8	0.0018	0.0051	0.0051	0.0002
9	0.0017	0.0054	0.0054	0.0002
10	0.0019	0.0055	0.0055	0.0002
11	0.0019	0.0052	0.0052	0.0002
12	0.0019	0.0052	0.0052	0.0002

#### 3.2.4 Kinetic evaluation of laccase-catalyzed reactions under batch conditions

Enzyme–substrate complex formation involves a simple reaction scheme with a reversible step and a dissociation step, creating a model for enzyme-catalyzed reactions. A key assumption in Michaelis–Menten kinetics is the rapid attainment of a steady state. Furthermore, the enzyme–substrate complex formation is treated as an equilibrium reaction, while the product formation step is irreversible.^48,49^

**Fig. 18:**
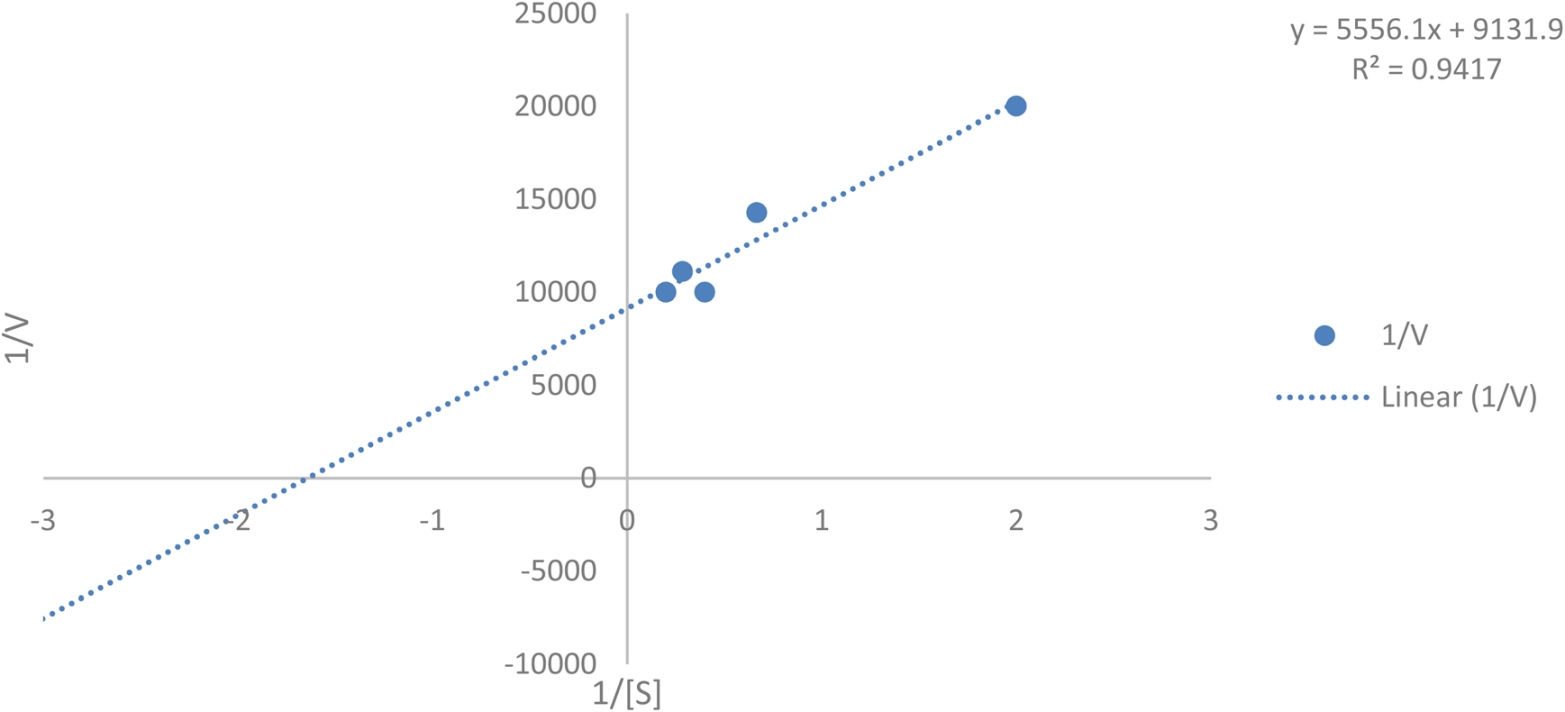
Lineweaver–Burk plot for laccase kinetics showing the linear relationship between 1/*V*_0_ and 1/[*S*], used to calculate *K*_m_ and *V*_max_.

Kinetic parameters were determined by measuring initial velocities (*V*_0_) at varying 5-HMF concentrations and plotting 1/*V*_0_*versus* 1/[*S*] in a Lineweaver–Burk plot (Fig. 18 below). The linear regression yielded the equation:1/*V*_0_ = 9131.9(1/[*S*]) + 5556.1 (*R*^2^ = 0.9417)From the *y*-intercept (1/*V*_max_ = 5556.1), the maximum velocity (*V*_max_) was calculated as 0.00011 mg mL^−1^ min^−1^. The Michaelis constant (*K*_m_) was derived using the relationship *K*_m_ = *V*_max_ × slope = 0.00011 × 5556.1 = 0.608428*K*_m = *V*_{max}\time\text{slope} = 0.00011\time 9131.9 = 0.608428*K*_m_ = *V*_max_ × slope = 0.00011 × 9131.9 = 0.608428 mM, indicating high substrate affinity. The strong linearity (*R*^2^ = 0.9417) confirms the applicability of the Michaelis–Menten model (Table 12).

**Table 12:** Enzyme kinetics of laccase for batch reaction

Sr. no.	[*S*]	Rate (*V*) (polynomial method)	1/*S*	1/*V*
1	0.5	0.00005	2	20 000
2	1.5	0.00007	0.666667	14 285.71
3	2.5	0.0001	0.4	10 000
4	3.5	0.00009	0.285714	11 111.11
5	5	0.0001	0.2	10 000

#### 3.2.5 Sustainable catalytic pathway for batch synthesis of FDCA from 5-HMF

The enhanced catalytic activity of the Co–Mn/AC catalyst can be attributed to the synergistic interaction between cobalt and manganese oxides, which promotes redox cycling and improves electron transfer during the oxidation process. The high surface area and porous structure of the activated carbon support ensure better dispersion of active metal sites and facilitate substrate accessibility. Furthermore, the incorporation of laccase in the hybrid system enhances substrate specificity and enables mild reaction conditions, minimizing side reactions. Together, these factors contribute to improved catalytic efficiency, selectivity, and recyclability of the system.

### 3.3 Characterization of the used catalyst (Co–Mn/AC)

**Fig. 19:**
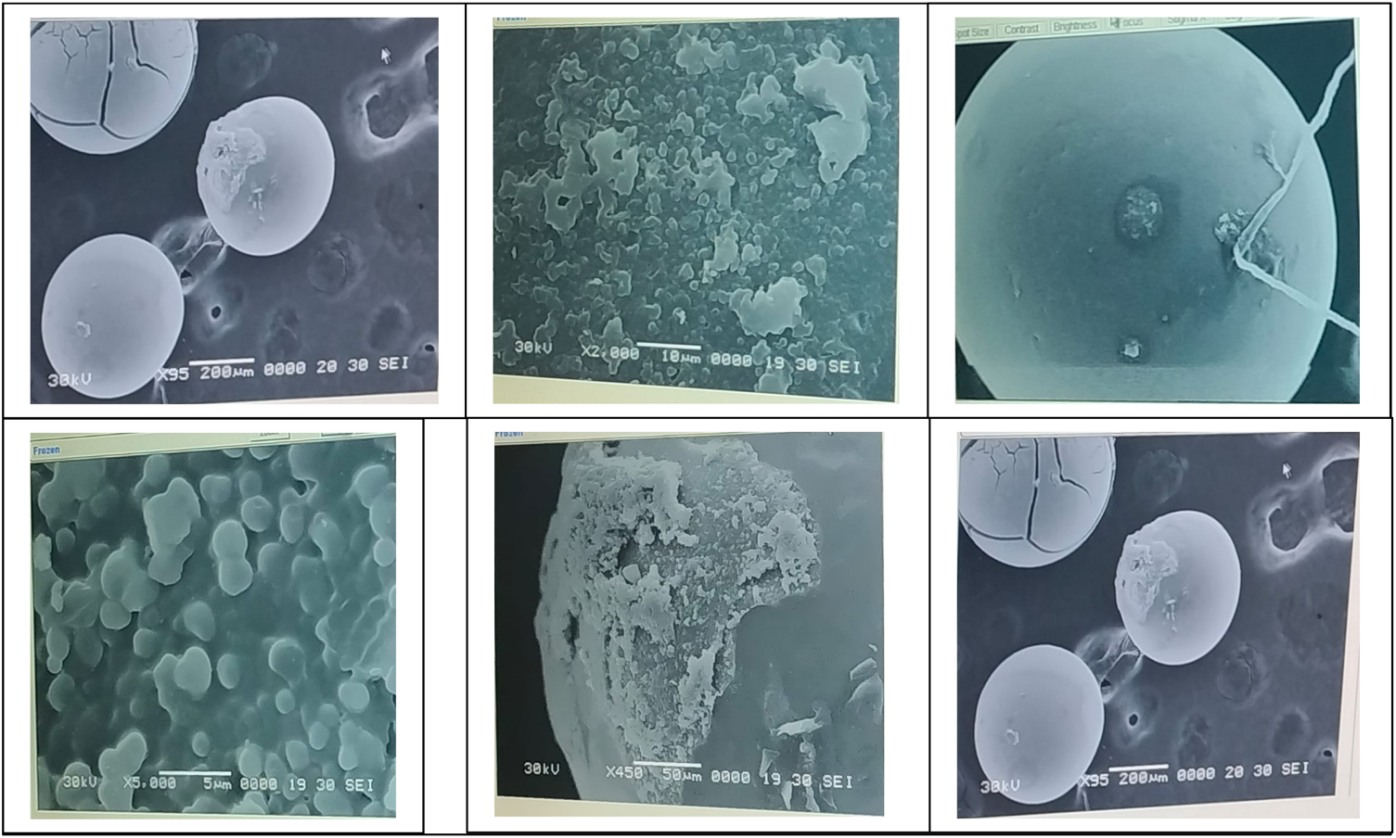
Scanning electron microscopy (SEM) image of the used Co–Mn/AC catalyst showing surface morphology.

Fig. 19 shows the Scanning Electron Microscopy (SEM) image of the used Co–Mn/AC catalyst. The micrograph reveals a porous and irregular surface morphology, which is characteristic of activated carbon-based supports. The presence of cobalt and manganese appears to contribute to the formation of dispersed metal clusters on the surface. After usage, the catalyst retains its structural integrity, although slight agglomeration and surface deposition may be observed, indicating possible coke formation or reaction-induced changes. These features suggest that the catalyst maintained good textural properties even after the reaction cycle, supporting its potential for reuse.

### 3.4 Effect of stirring rate on catalytic conversion efficiency

**Fig. 20:**
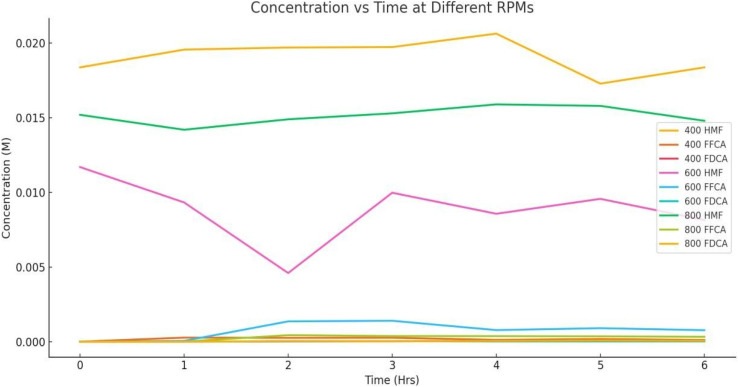
Effect of agitation speed, at 400, 600, and 800 rpm.

The conversion of 5-hydroxymethylfurfural (5-HMF) into 2,5-furandicarboxylic acid (FDCA) *via* catalytic oxidation is a promising pathway for producing sustainable bio-based chemicals. Among the various factors influencing the efficiency of this oxidation process, agitation speed plays a vital role in regulating mass transfer, oxygen availability, and interaction between the reactant and catalyst^50^ (MnO_2_ and Co–Mn/AC). To explore this effect, a series of experiments were carried out at a constant temperature of 90 °C, under 3 bar of air pressure, using 1 mmol of 5-HMF and 0.1 g of Co–Mn/AC catalyst in 70 mL of water.^51,52^ The stirring speeds investigated were 400, 600, and 800 rpm, and concentrations of 5-HMF, the intermediate 2,5-formylfurancarboxylic acid (FFCA), and the final product FDCA were measured over a 6-hour reaction period. The experimental data presented in Table 13 and Fig. 20 reflect how varying agitation intensity influences not only the rate of 5-HMF depletion but also the accumulation of FFCA and FDCA. Since oxygen solubility and catalyst–substrate contact are strongly dependent on agitation, analyzing the impact of stirring speeds provides key insights into mass transfer limitations, intermediate dynamics, and bottlenecks in the final oxidation step. This section critically evaluates how different stirring rates affect substrate consumption and product formation, and it provides a foundational understanding for optimizing reaction conditions to improve FDCA yields in aqueous-phase oxidation systems.^53,54^

**Table 13:** Effect of agitation speed, at 400, 600, and 800 rpm

Time	400 rpm			600 rpm			800 rpm		
	Conc (HMF)	Conc (FFCA)	Con (FDCA)	Con (HMF)	Con (FFCA)	Con (FDCA)	Con (HMF)	Con (FFCA)	Con (FDCA)
0	0.01838	0	0	0.0117	0	0	0.0152	0	0
1	0.01957	0.000270967	0	0.00933	0.0000527	0	0.0142	0	0
2	0.01971	0.000256822	0.000018365	0.00460	0.00136	0.0000243919	0.0149	0.00043709	0.0000436115
3	0.01974	0.000262079	0.0000258428	0.00998	0.00140	0.0000319339	0.0153	0.00037559	0.0000256725
4	0.02064	0.000120898	0.0000481358	0.00857	0.000775	0.000016865	0.0159	0.00037233	0.0000529907
5	0.01729	0.000176306	0.0000359592	0.00957	0.000903	0.0000166006	0.0158	0.00035633	0.0000761545
6	0.01838	0.000113072	0.000033592	0.00815	0.000769	0.0000186833	0.0148	0.00032744	0.0000621584

The results presented in Table 13 and Fig. 20 demonstrate the significant influence of agitation speed on the catalytic oxidation of 5-HMF to FDCA. At the lowest stirring speed of 400 rpm, 5-HMF showed incomplete conversion, with its concentration fluctuating from an initial 0.0117 M to 0.00815 M at hour 6. FFCA, the oxidation intermediate, reached a peak of 0.0014 M at hour 3, but its slow conversion to FDCA limited the final product concentration, which peaked at only 3.19 × 10^−5^ M. This behavior suggests that lower agitation restricts oxygen transfer and reduces catalyst–reactant interaction, leading to mass transfer limitations. A moderate increase in stirring speed to 600 rpm showed a similar trend but slightly improved 5-HMF conversion, as evidenced by its lower final concentration of 0.0076 M. FFCA still accumulated and declined over time, peaking again at hour 3, while FDCA formation remained modest, highlighting a kinetic barrier in the final oxidation step. At 800 rpm, where mixing and oxygen availability are significantly enhanced, FDCA formation improved, peaking at 7.62 × 10^−5^ M by hour 5, while FFCA concentrations were lower compared to slower stirring rates, indicating better progression through the intermediate stage. Although 5-HMF consumption was less dramatic at this speed, the more efficient conversion to FDCA suggests that enhanced agitation accelerates catalytic oxidation beyond the FFCA stage. These observations give information about the importance of optimizing agitation to address both mass transfer and reaction kinetics and underscore that while increased stirring improves performance, full conversion to FDCA still requires further improvements in catalyst efficiency and process parameters.

### 3.5 Catalyst loading-dependent performance in the oxidation of 5-HMF to FDCA

**Fig. 21:**
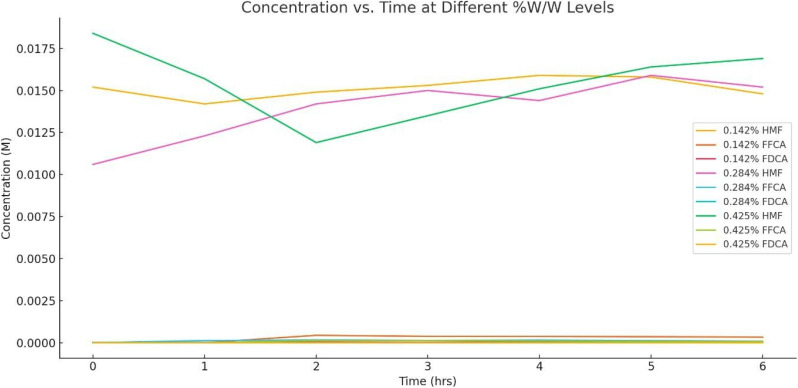
Effect of catalyst loading, at 0.142, 0.284, and 0.425% W/W.

The 2,5-furandicarboxylic acid (FDCA) has emerged as a pivotal bio-based monomer, primarily synthesized through the catalytic oxidation of 5-hydroxymethylfurfural (5-HMF). Among the numerous catalytic systems investigated, bimetallic catalysts such as cobalt–manganese supported on activated carbon (Co–Mn/Ac) have been tried in converting 5-HMF into FDCA.^55,56^ A critical factor influencing the efficiency of this catalytic transformation is the concentration of the catalyst used during the reaction. To elucidate the impact of catalyst loading on the conversion efficiency and product distribution, a series of experiments were conducted using three different catalyst concentrations: 0.142%, 0.284%, and 0.425% w/w. The initial concentration of 5-HMF was maintained at 0.1 M, while reaction conditions, including temperature (90 °C), pressure (3 bar air), stirring rate (800 rpm), and sodium bicarbonate addition (0.084 g), were held constant.^57,58^ This controlled environment allowed for a clear comparative analysis of the catalytic activity at varying concentrations. Fig. 21 and Table 14 present the temporal evolution of reactant (5-HMF), intermediate (2,5-formylfurancarboxylic acid or FFCA), and product (FDCA) concentrations over a 6-hour period for each catalyst loading. These data are crucial in understanding how catalyst concentration affects not only the rate of 5-HMF consumption but also the accumulation and transformation of key intermediates, thereby determining the overall yield of FDCA.^59^

**Table 14:** Effect of catalyst loading, at 0.142, 0.284, and 0.425% W/W

Time	0.142% W/W			0.284% W/W			0.425% W/W		
	Conc (5- HMF)	Conc (FFCA)	Con (FDCA)	Con (5-HMF)	Con (FFCA)	Con (FDCA)	Con (5-HMF)	Con (FFCA)	Con (FDCA)
0	0.0152	0	0	0.0106	0	0	0.0184	0	0
1	0.0142	0	0	0.0123	0.000123	0	0.0157	0	0
2	0.0149	0.000437	0.0000436	0.0142	0.000159	0	0.0119	0.000126	0
3	0.0153	0.000376	0.0000257	0.0150	0.000133	0	0.0135	0.000132	0
4	0.0159	0.000372	0.000053	0.0144	0.000149	0	0.0151	0.0000961	0
5	0.0158	0.00035	0.0000762	0.0159	0.000126	0.0000136	0.0164	0.0000803	0
6	0.0148	0.000327	0.0000622	0.0152	0.0000852	0.0000907	0.0169	0.0000631	0

The data presented in Fig. 21 and Table 14 reveal a complex interplay between catalyst concentration and the conversion of 5-HMF to FDCA. At the lowest catalyst loading (0.142% w/w), the system demonstrates steady conversion of 5-HMF with gradual accumulation of FFCA and formation of FDCA, reaching a peak FDCA concentration of 7.62 × 10^−5^ M by hour 5. This indicates that the catalytic oxidation is relatively slow. Increasing the catalyst concentration to 0.284% does not significantly enhance the overall FDCA yield; in fact, FDCA formation remains limited, with a peak of only 1.36 × 10^−5^ M. This could be attributed to the evaporation of water at elevated temperatures, leading to fluctuations in reactant concentrations and potentially hindering complete oxidation. At the highest catalyst loading (0.425%), a more pronounced initial drop in 5-HMF concentration is observed, suggesting more efficient initial oxidation. However, neither FFCA nor FDCA accumulates substantially, and FDCA remains undetected throughout the 6-hour period. This may be the deactivation of active sites at higher catalyst concentrations, preventing the final oxidation step. These results suggest that simply increasing catalyst loading is not sufficient to improve FDCA yields; rather, optimization of other parameters, such as solvent stability, oxygen availability, or reaction time, may be essential. Thus, while Co–Mn/Ac catalysts show potential for FDCA production, the system requires further refinement to enhance the selectivity and efficiency of the complete oxidation pathway from 5-HMF to FDCA.

### 3.6 Impact of reaction pressure on yield and selectivity in batch oxidation systems

The pressure plays a crucial role by regulating oxygen solubility in the reaction medium, directly impacting the oxidation rates of 5-HMF and its intermediates. Understanding the effect of pressure is essential to optimizing the reaction for higher FDCA yields and improving the overall sustainability and economic feasibility of the process. This study evaluates the effect of varying pressures (1, 2, and 3 bar) on the conversion efficiency of 5-HMF to FDCA.^60^

**Fig. 22:**
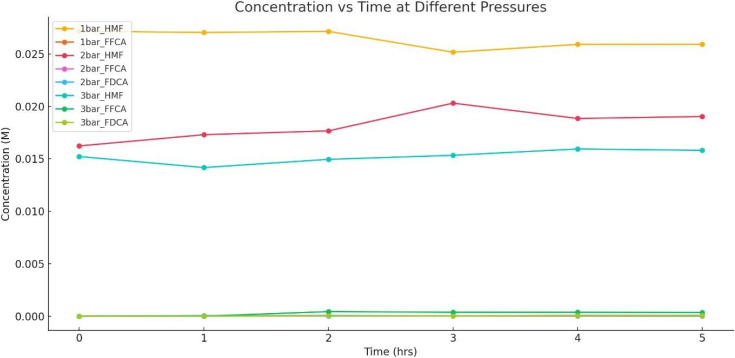
Effect of pressure at 1, 2, and 3 bar.

The conversion of 5-HMF to FDCA is strongly influenced by air pressure, as depicted in Table 15 and Fig. 22. At 1 bar, minimal substrate consumption and no FDCA formation were observed, indicating oxygen limitations that restricted oxidation efficiency. The FFCA intermediate accumulated in trace amounts (0.0000178 M at hour 3), but the lack of FDCA formation suggests an incomplete oxidation pathway. Increasing the pressure to 2 bar resulted in slightly enhanced oxidation, with FDCA peaking at 0.0000594 M at hour 1, though fluctuations in 5-HMF concentration indicate incomplete conversion. The highest pressure of 3 bar exhibited the best oxidation efficiency, with FDCA formation reaching 0.0000762 M.

**Table 15:** Effect of pressure at 1,2, and 3 bar

Time	1 bar		2 bar			3 bar		
	Conc (5-HMF)	Conc (FFCA)	Con (5-HMF)	Con (FFCA)	Con (FDCA)	Con (5-HMF)	Con (FFCA)	Con (FDCA)
0	0.027163	0	0.016226	0	0	0.015219	0	0
1	0.027039	0.0000163	0.017299	0.0000442	0.0000594	0.014167	0	0
2	0.027146	0.0000172	0.017663	0.0000663	0.0000165	0.014949	0.000437	0.0000436
3	0.02516	0.0000178	0.020308	0.0000352	0.000038	0.015336	0.000376	0.0000257
4	0.025909	0.0000104	0.018845	0.0000719	0.0000317	0.015938	0.000372	0.000053
5	0.025909	0.0000103	0.019031	0.0000515	0.0000255	0.01581	0.000356	0.0000762

### 3.7 Catalyst reusability test

**Fig. 23:**
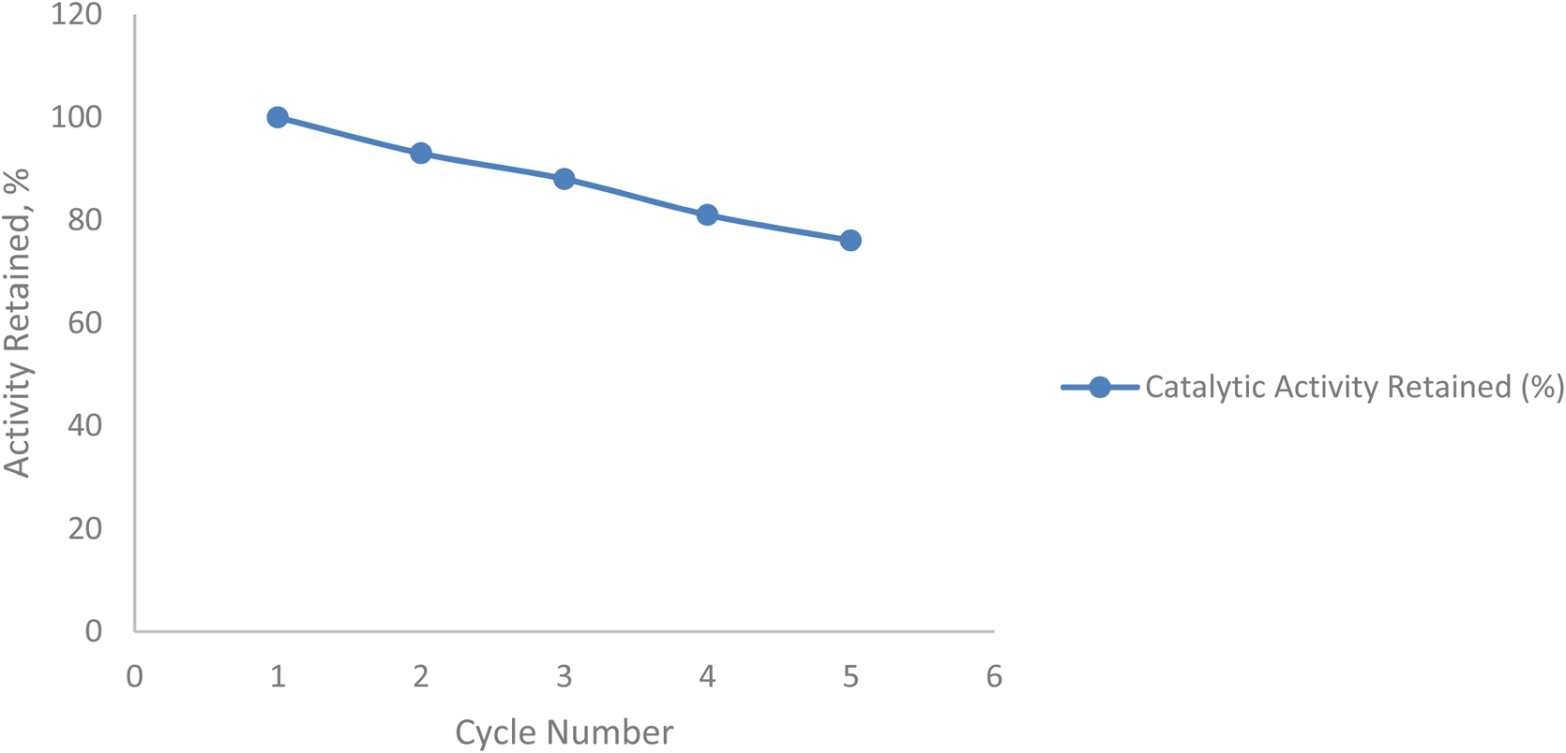
Reusability profile of the hybrid catalyst (Co–Mn/AC + immobilized laccase) over five cycles.

Fig. 23 and Table 16 show the reusability performance of the hybrid catalyst (Co–Mn/AC + immobilized laccase) over five consecutive cycles. A gradual decrease in catalytic activity was observed, with the catalyst retaining approximately 76% of its initial activity after the fifth cycle. This decline is attributed to partial enzyme deactivation, metal leaching, and surface fouling during repeated use.

**Table 16:** Catalytic activity retained by the hybrid catalyst across successive reaction cycles

Cycle number (*n*)	Catalytic activity retained (%)
1	100
2	93
3	88
4	81
5	76

## 4 Conclusion

In summary, this study presents a novel, environmentally sustainable biocatalytic protocol for the oxidative conversion of 5-hydroxymethylfurfural (5-HMF) to 2,5-furandicarboxylic acid (FDCA) using laccase as a green catalyst. The initial batch reactions, conducted with 5 mM of 5-HMF and 1 mg mL^−1^ of laccase enzyme in an acetate buffer system, yielded promising results. Process parameters were systematically optimized to enhance both substrate conversion and product selectivity, with the optimal conditions identified as 40 °C, pH 5, and 1 mg mL^−1^ enzyme concentration. Under these conditions, the laccase enzyme exhibited significant catalytic efficiency in the oxidation of 5-HMF, demonstrating strong potential as an eco-friendly biocatalyst.

A key novelty of this study lies in its integration of immobilized enzyme systems on functionalized glass beads, enabling reuse and operational stability while aligning with principles of green chemistry. Though continuous flow reactions predominantly led to the accumulation of 2,5-diformylfuran (DFF) due to the fast conversion of 5-HMF, the pathway to FDCA remained viable with extended residence times. This highlights the system's potential for modular scalability and time-resolved process control.

Importantly, the entire approach avoids the use of harsh chemicals or heavy metal catalysts, minimizing environmental impact and supporting the transition toward sustainable chemical manufacturing. The study not only reinforces the utility of enzymatic catalysis in biomass valorization but also introduces a clean, scalable strategy for converting bio-based intermediates into high-value monomers like FDCA, positioning it as a promising alternative to petroleum-derived pathways within the circular bioeconomy.

## Author contributions

Rakesh Gujar (corresponding author): conceptualization, methodology, investigation, data curation, formal analysis, writing – original draft preparation, supervision, and correspondence handling. Raju Thombe: project administration, writing – review & editing. Pratibha Dhindale: validation, experimental support, visualization, writing – review & editing.

## Conflicts of interest

There is no conflict of interest.

## Supplementary Material

RA-015-D5RA04438C-s001

## Data Availability

All data supporting the findings of this study are available within the article and its SI files. No additional datasets or code were deposited in external repositories. Supplementary information: Detailed experimental procedures, additional HPLC spectra, as well as supporting tables and figures that provide extended data in support of the main manuscript results. See DOI: https://doi.org/10.1039/d5ra04438c.
