# Supplementary material for: Selective and green conversion of 5-HMF to FDCA via enzymatic (laccase) and transition metal (MnO2 and Co–Mn/AC) catalysis in an integrated system
Source: RSC Adv. 2025 Sep 8;15(39):32337–60. doi: 10.1039/d5ra04438c (PMC12415952; doi:10.1039/d5ra04438c)
Supplement: RA-015-D5RA04438C-s001 [file RA-015-D5RA04438C-s001.pdf]

This journal is © The Royal Society of Chemistry 2025

Supplementary Information

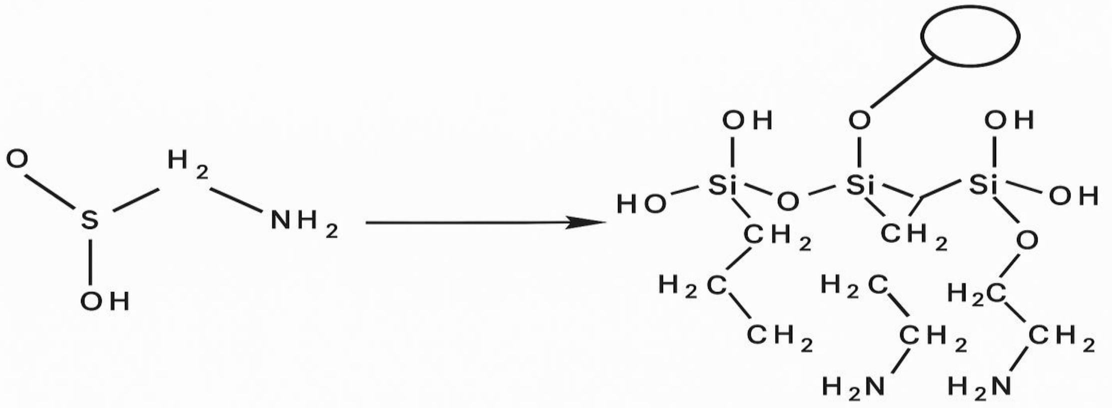

3- aminopropyltriethoxysilane

Stable Glass Beads of Siloxane

Figure 1: Schematic Representation of Laccase Immobilization on APTES-Functionalized Glass Beads

Effect of Substrate Concentration on Product Formation and Catalytic Efficiency

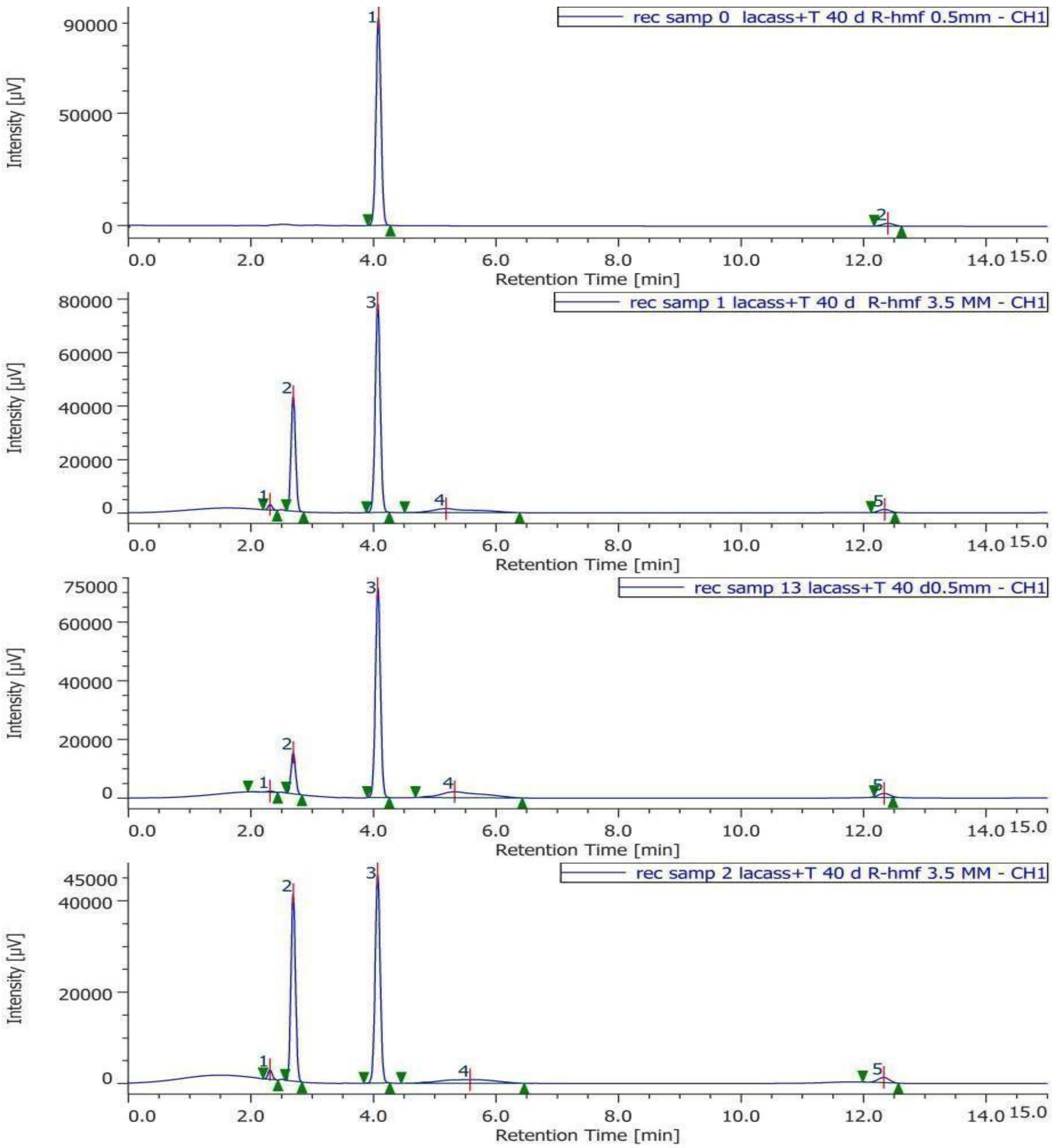

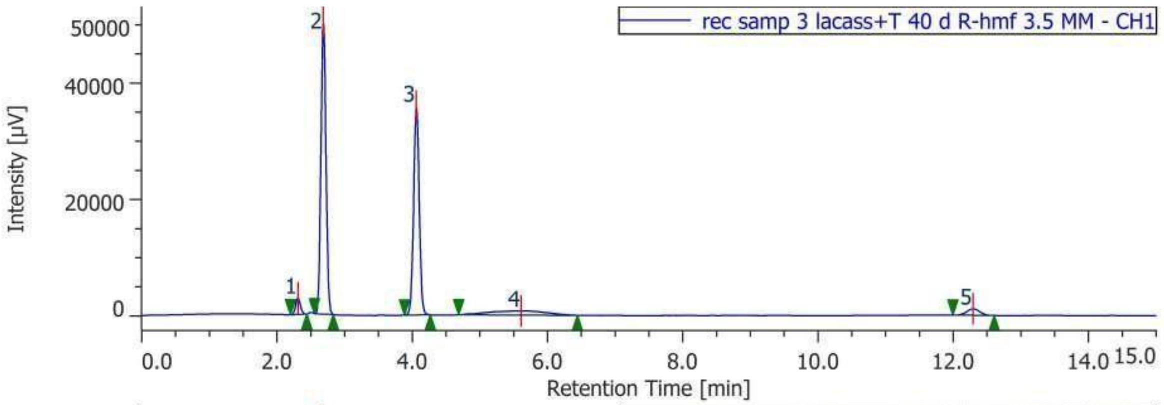
rec samp 3 lacass+T 40 d R-hmf 3.5 MM - CH1
Intensity [µV]
50000
40000
20000
0
1
2
3
4
5
0.0
2.0
4.0
6.0
8.0
10.0
12.0
14.0
15.0
Retention Time [min]

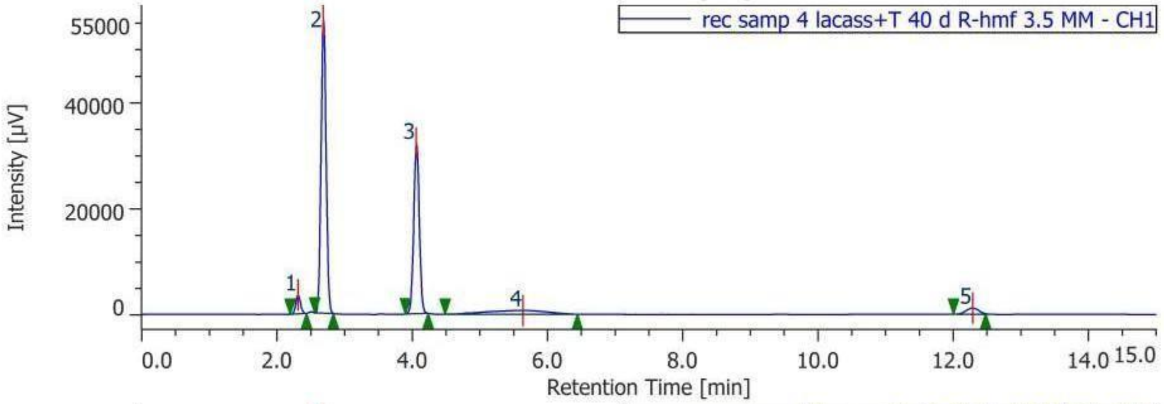
rec samp 4 lacass+T 40 d R-hmf 3.5 MM - CH1
Intensity [µV]
55000
40000
20000
0
1
2
3
4
5
0.0
2.0
4.0
6.0
8.0
10.0
12.0
14.0
15.0
Retention Time [min]

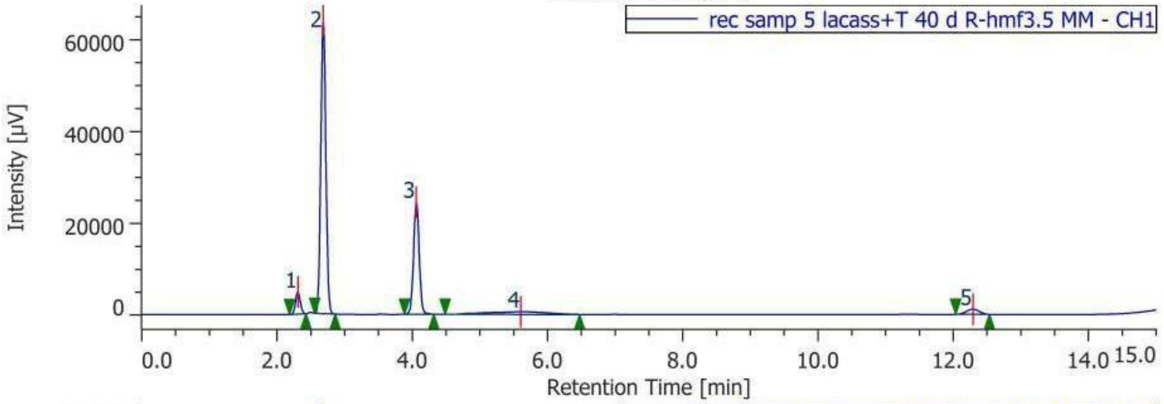
rec samp 5 lacass+T 40 d R-hmf3.5 MM - CH1
Intensity [µV]
60000
40000
20000
0
1
2
3
4
5
0.0
2.0
4.0
6.0
8.0
10.0
12.0
14.0
15.0
Retention Time [min]

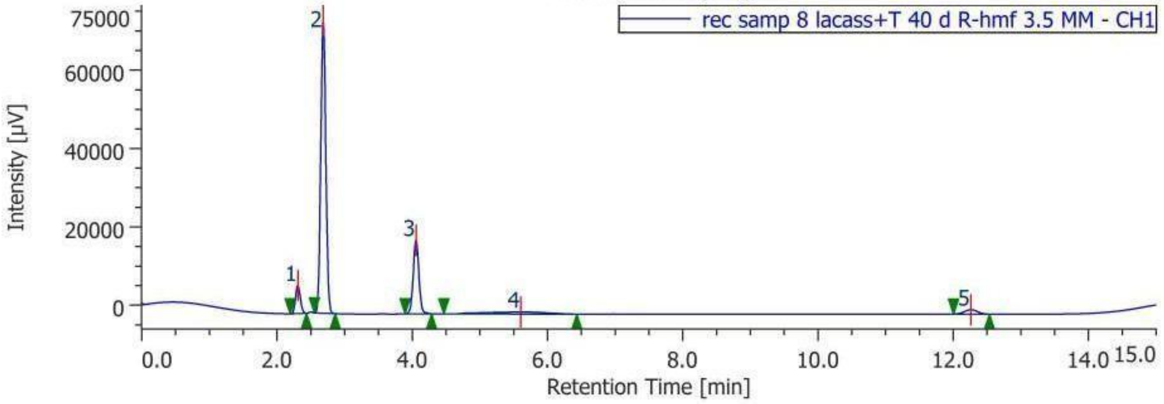
rec samp 8 lacass+T 40 d R-hmf 3.5 MM - CH1
Intensity [µV]
75000
60000
40000
20000
0
1
2
3
4
5
0.0
2.0
4.0
6.0
8.0
10.0
12.0
14.0
15.0
Retention Time [min]

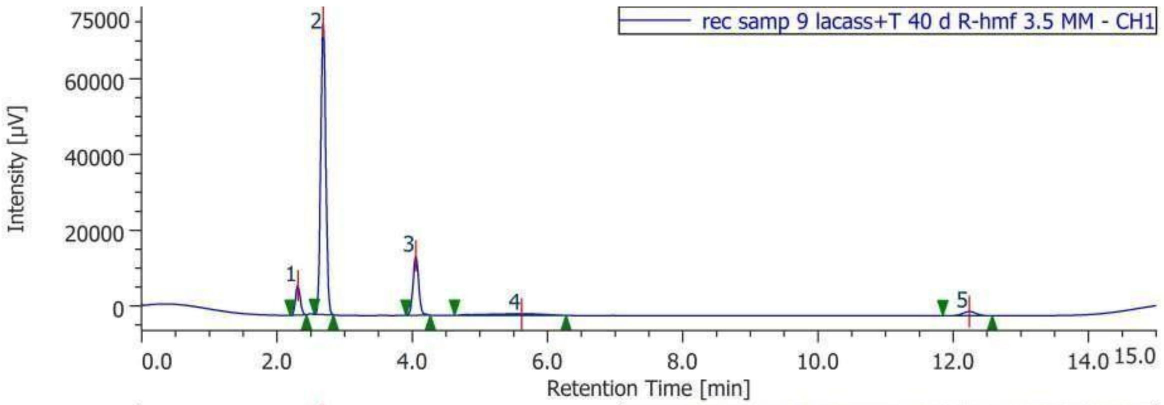
rec samp 9 lacass+T 40 d R-hmf 3.5 MM - CH1
Intensity [µV]
Retention Time [min]
75000
60000
40000
20000
0
0.0
2.0
4.0
6.0
8.0
10.0
12.0
14.0
15.0
1
2
3
4
5

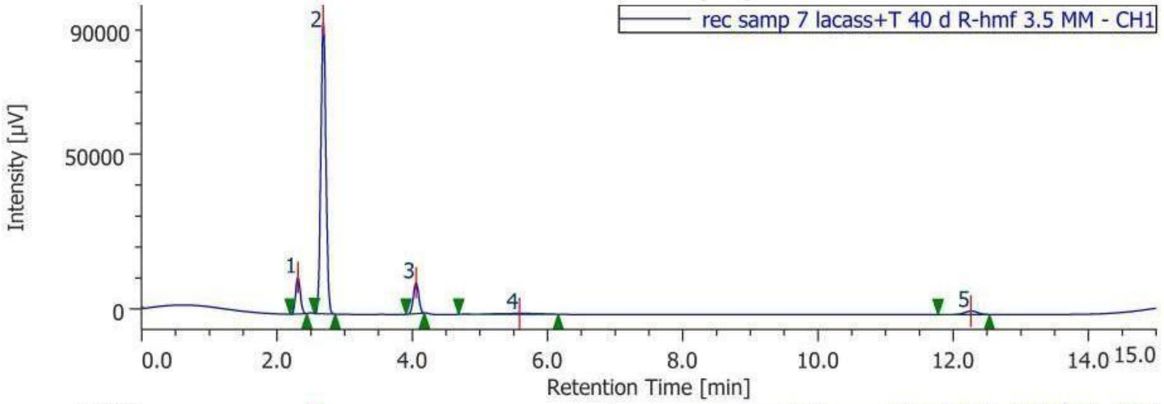
rec samp 7 lacass+T 40 d R-hmf 3.5 MM - CH1
Intensity [µV]
Retention Time [min]
90000
50000
0
0.0
2.0
4.0
6.0
8.0
10.0
12.0
14.0
15.0
1
2
3
4
5

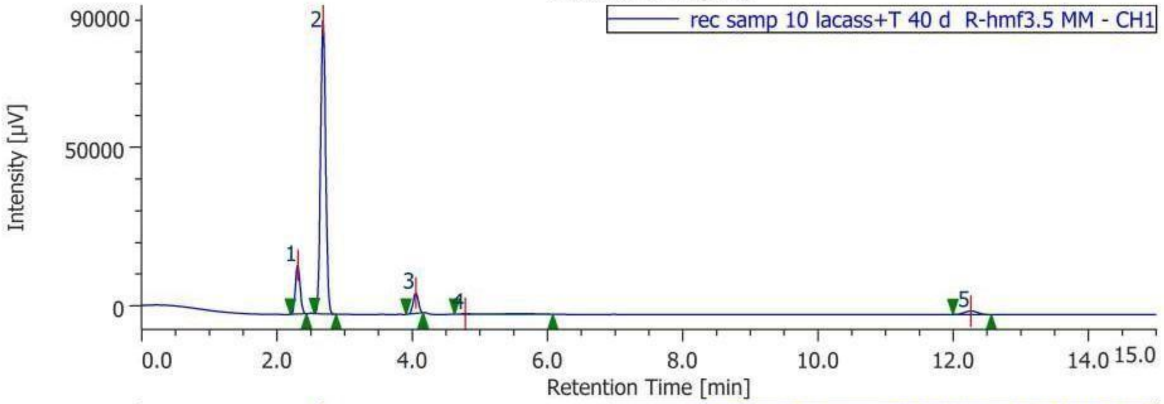
rec samp 10 lacass+T 40 d R-hmf3.5 MM - CH1
Intensity [µV]
Retention Time [min]
90000
50000
0
0.0
2.0
4.0
6.0
8.0
10.0
12.0
14.0
15.0
1
2
3
4
5

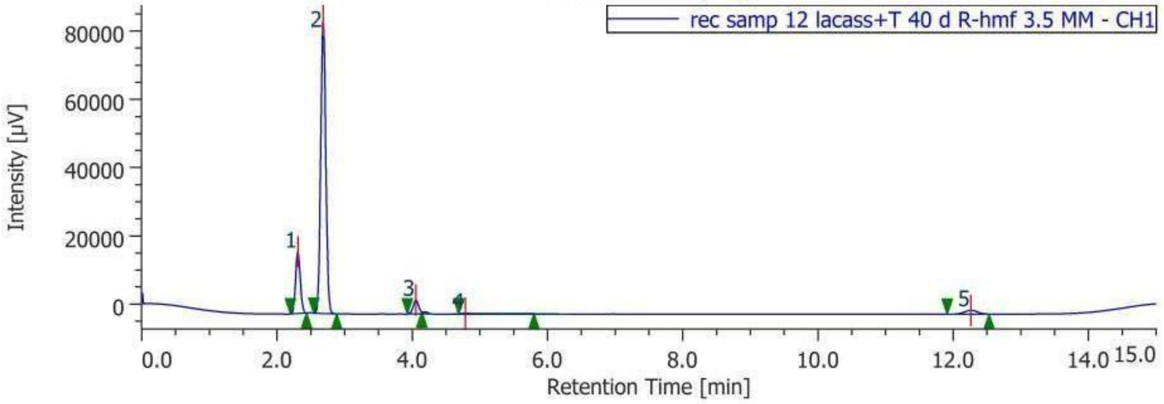
rec samp 12 lacass+T 40 d R-hmf 3.5 MM - CH1
Intensity [µV]
Retention Time [min]
80000
60000
40000
20000
0
0.0
2.0
4.0
6.0
8.0
10.0
12.0
14.0
15.0
1
2
3
4
5

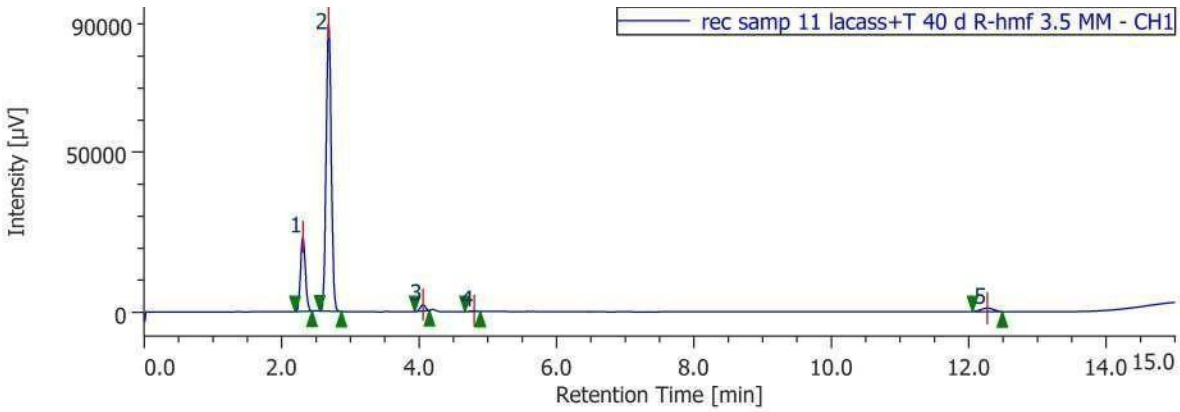

Figure 2: HPLC Chromatogram Showing 5-HMF Peaks from 0 h to 12h for 25 to 250 mM Substrate Concentrations (1-FDCA, 2-HFCA, 3-HMF, 4-DFF, 5-Phenol [Internal Standard])

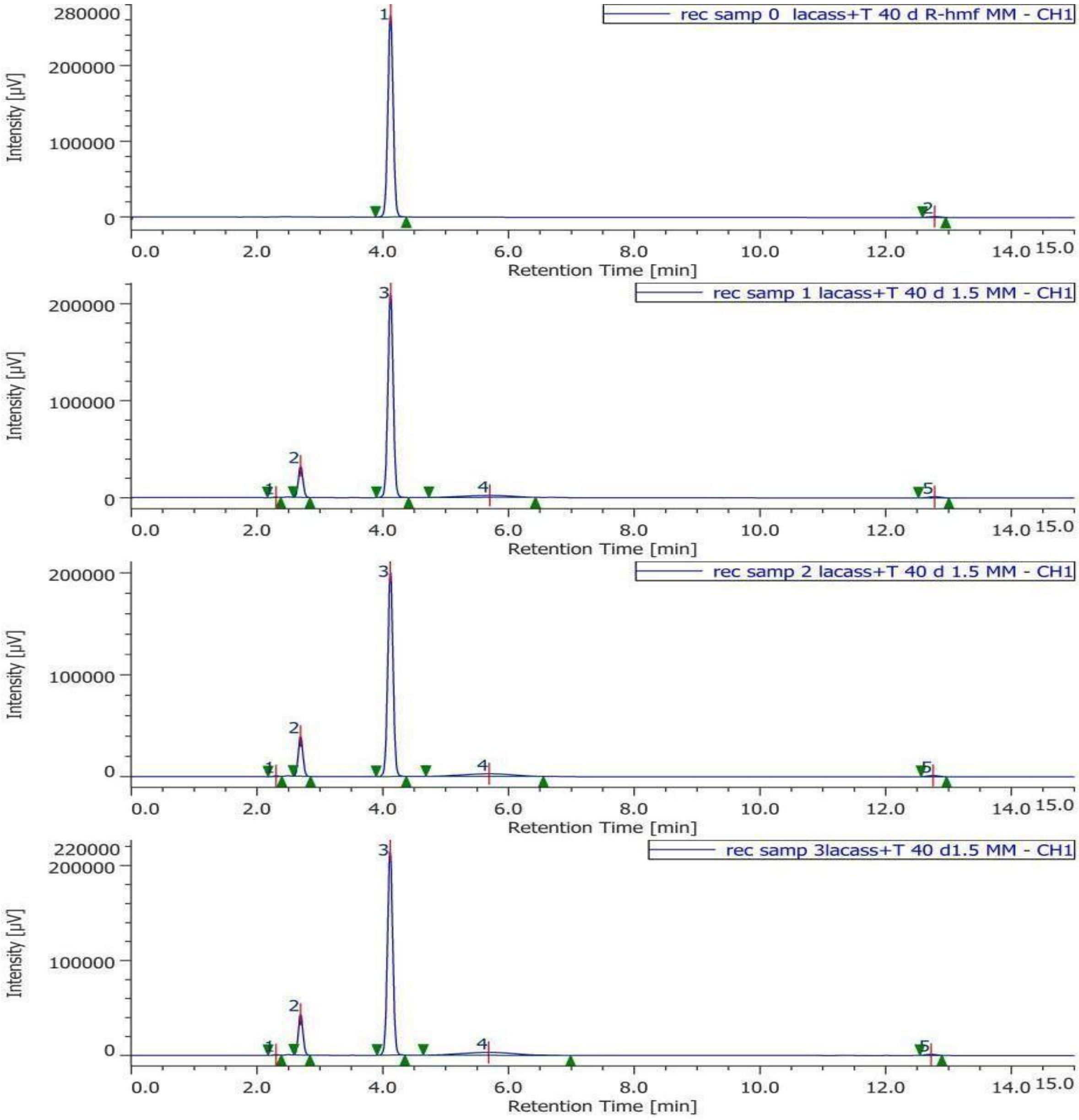

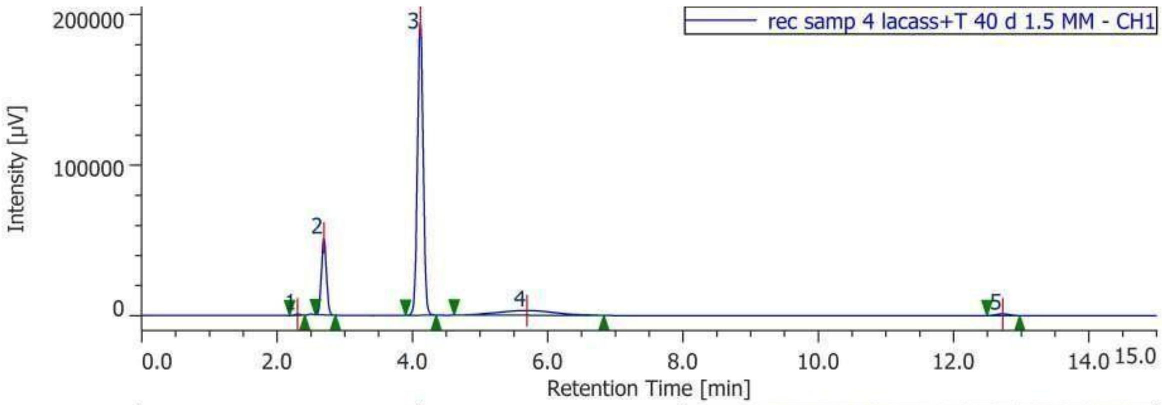
rec samp 4 lacass+T 40 d 1.5 MM - CH1
Intensity [µV]
200000
100000
0
Retention Time [min]
0.0
2.0
4.0
6.0
8.0
10.0
12.0
14.0
15.0

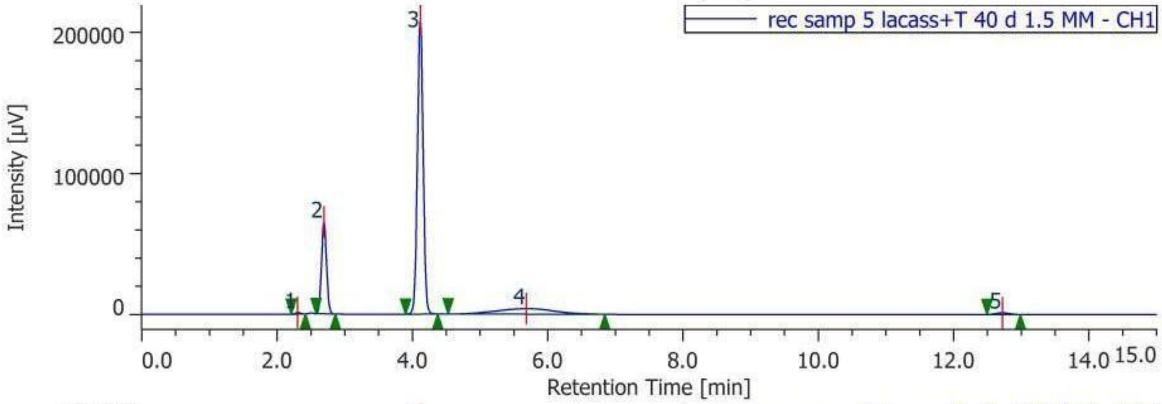
rec samp 5 lacass+T 40 d 1.5 MM - CH1
Intensity [µV]
200000
100000
0
Retention Time [min]
0.0
2.0
4.0
6.0
8.0
10.0
12.0
14.0
15.0

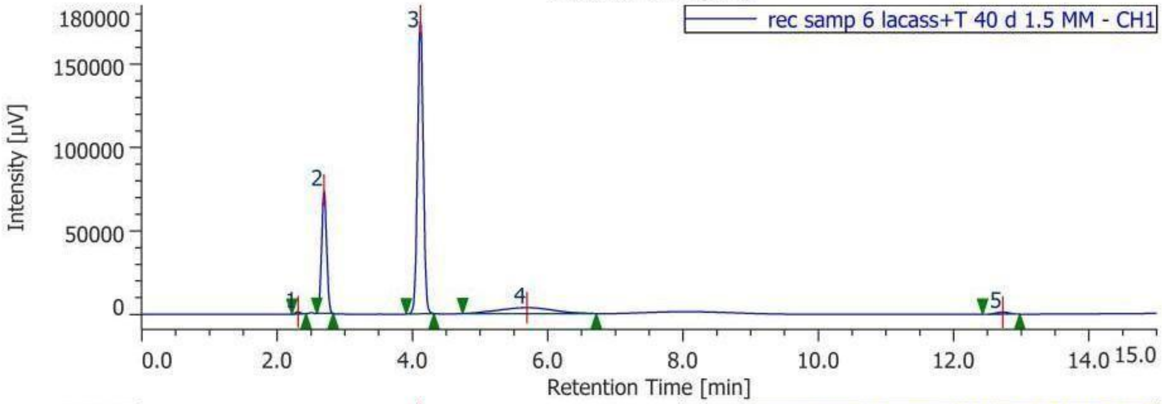
rec samp 6 lacass+T 40 d 1.5 MM - CH1
Intensity [µV]
180000
150000
100000
50000
0
Retention Time [min]
0.0
2.0
4.0
6.0
8.0
10.0
12.0
14.0
15.0

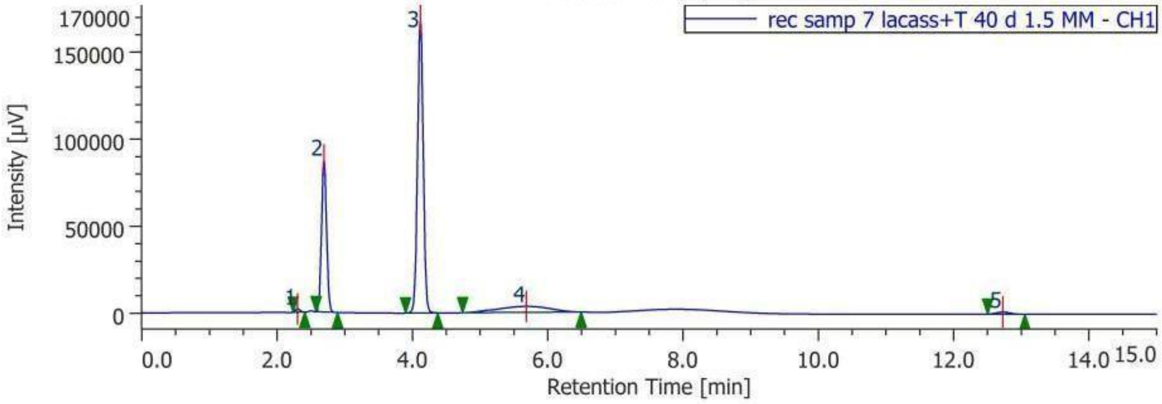
rec samp 7 lacass+T 40 d 1.5 MM - CH1
Intensity [µV]
170000
150000
100000
50000
0
Retention Time [min]
0.0
2.0
4.0
6.0
8.0
10.0
12.0
14.0
15.0

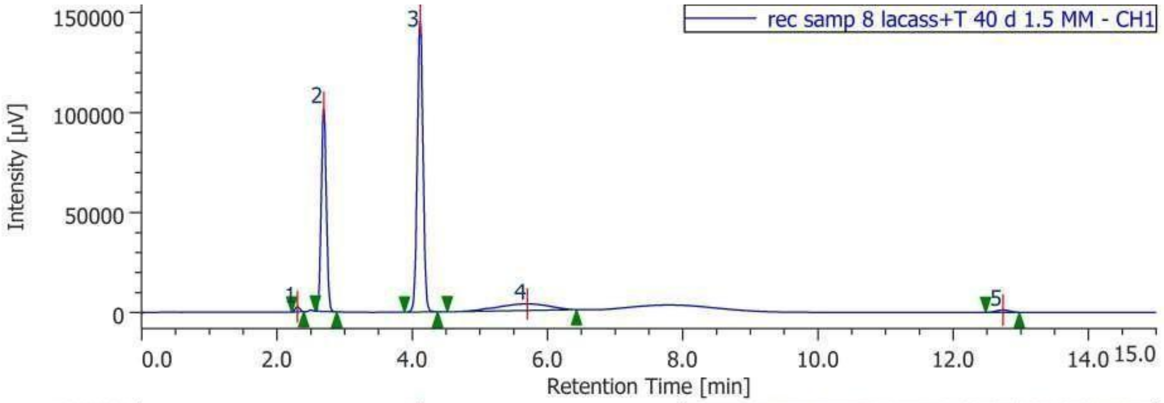

rec samp 8 lacass+T 40 d 1.5 MM - CH1
Intensity [µV]
150000
100000
50000
0
1
2
3
4
5
0.0
2.0
4.0
6.0
8.0
10.0
12.0
14.0
15.0
Retention Time [min]

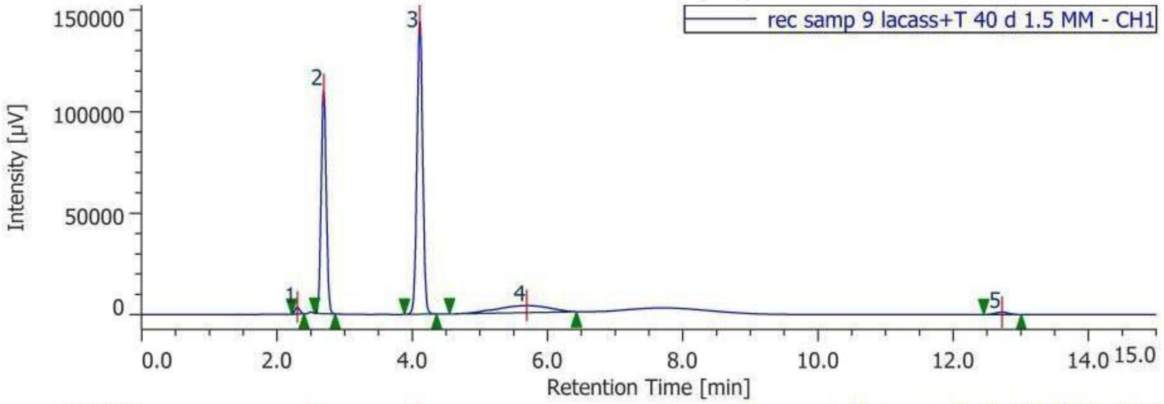

rec samp 9 lacass+T 40 d 1.5 MM - CH1
Intensity [µV]
150000
100000
50000
0
1
2
3
4
5
0.0
2.0
4.0
6.0
8.0
10.0
12.0
14.0
15.0
Retention Time [min]

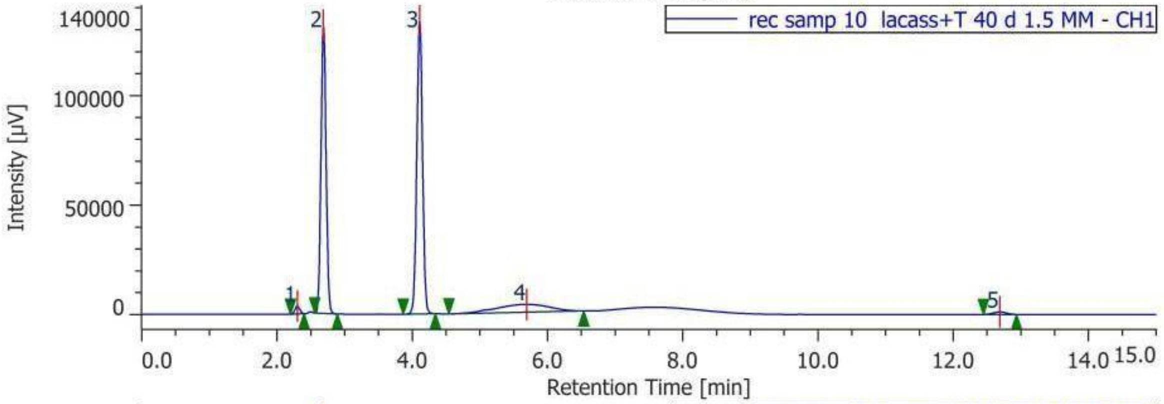

rec samp 10 lacass+T 40 d 1.5 MM - CH1
Intensity [µV]
140000
100000
50000
0
1
2
3
4
5
0.0
2.0
4.0
6.0
8.0
10.0
12.0
14.0
15.0
Retention Time [min]

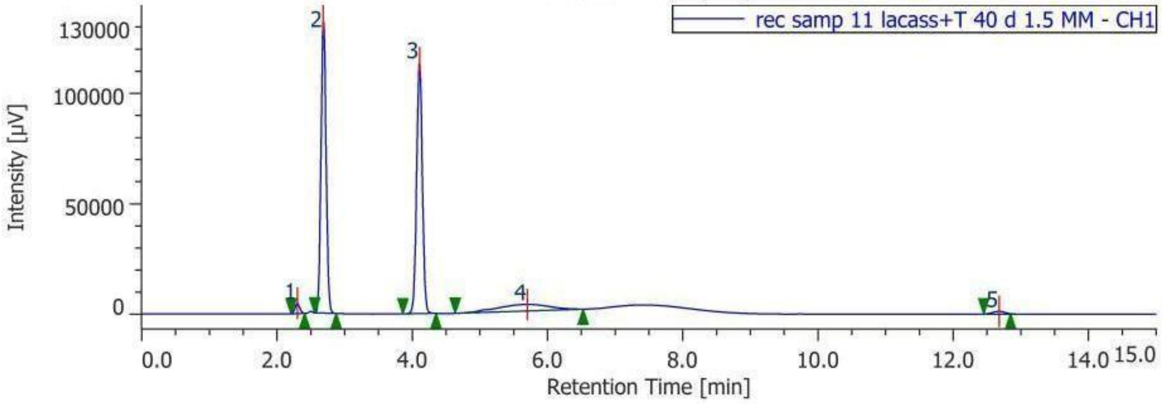

rec samp 11 lacass+T 40 d 1.5 MM - CH1
Intensity [µV]
130000
100000
50000
0
1
2
3
4
5
0.0
2.0
4.0
6.0
8.0
10.0
12.0
14.0
15.0
Retention Time [min]

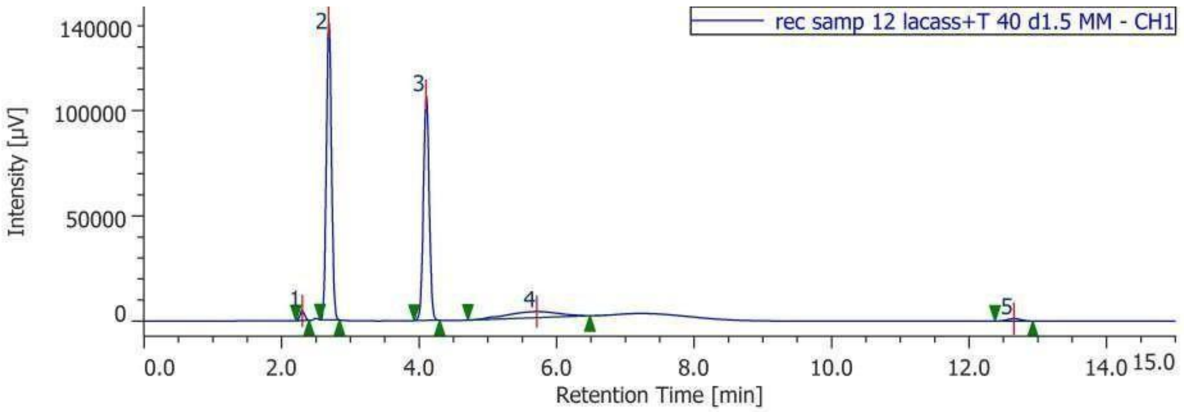

Figure 3: HPLC Chromatogram Showing 2,5-diformylfuran Peaks from 0 h to 12h for 25 to 250 mM Substrate Concentrations (1-FDCA, 2-HFCA, 3-HMF, 4-DFF, 5-Phenol [Internal Standard])

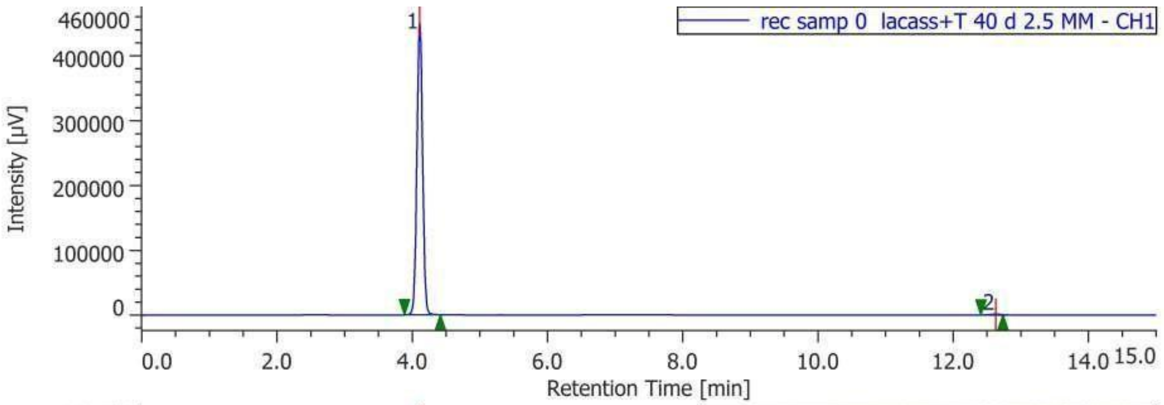
rec samp 0 lacass+T 40 d 2.5 MM - CH1
Intensity [µV]
Retention Time [min]

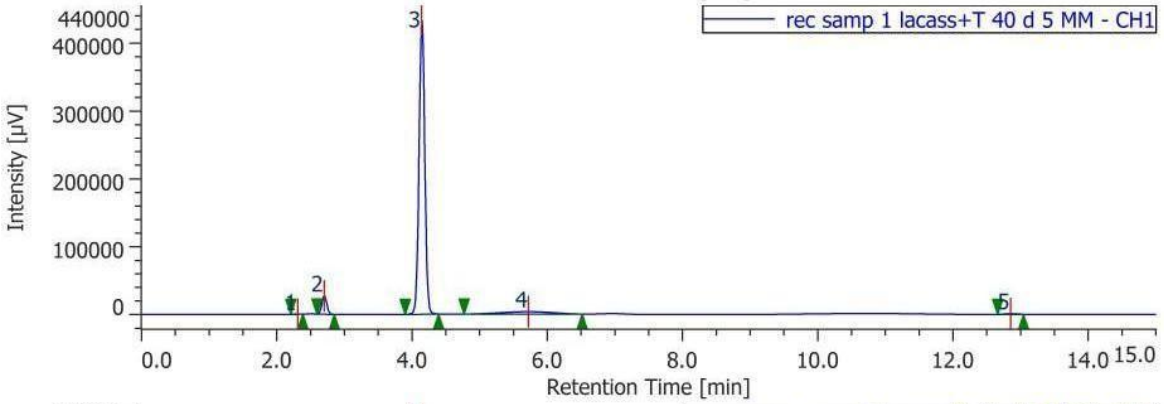
rec samp 1 lacass+T 40 d 5 MM - CH1
Intensity [µV]
Retention Time [min]

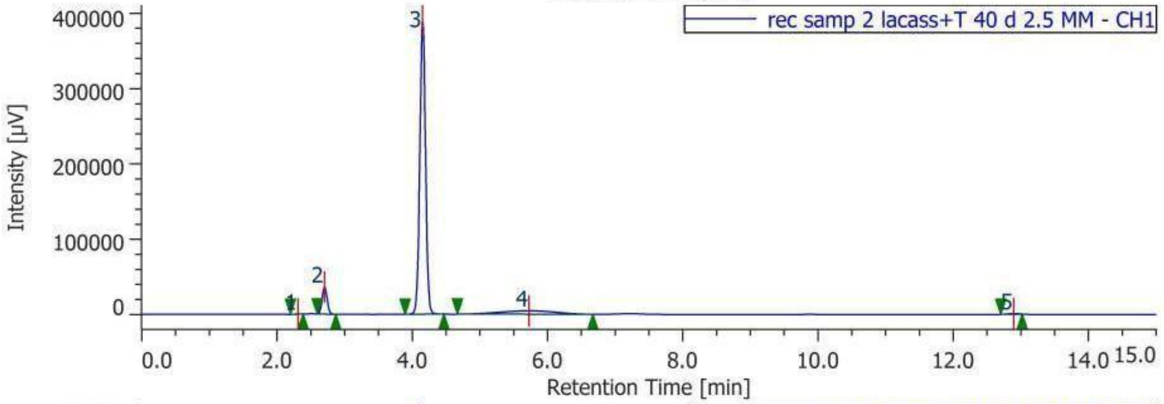
rec samp 2 lacass+T 40 d 2.5 MM - CH1
Intensity [µV]
Retention Time [min]

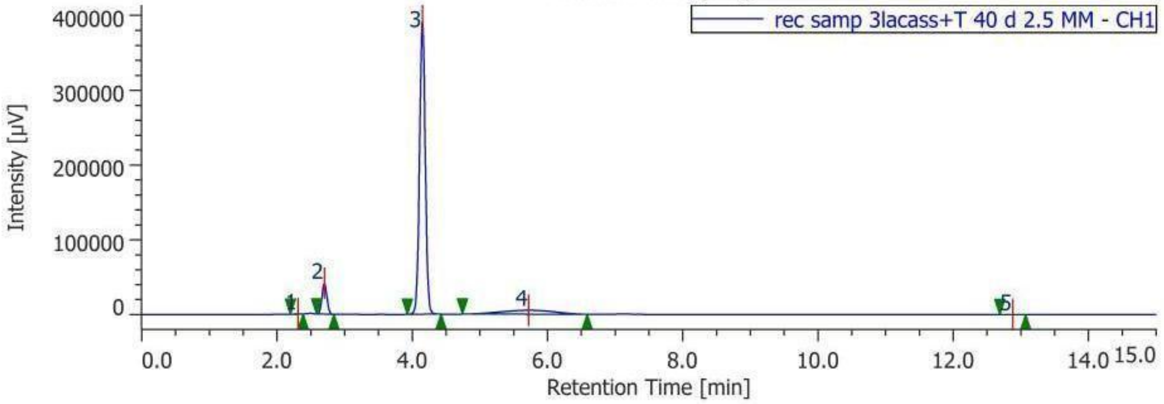
rec samp 3lacass+T 40 d 2.5 MM - CH1
Intensity [µV]
Retention Time [min]

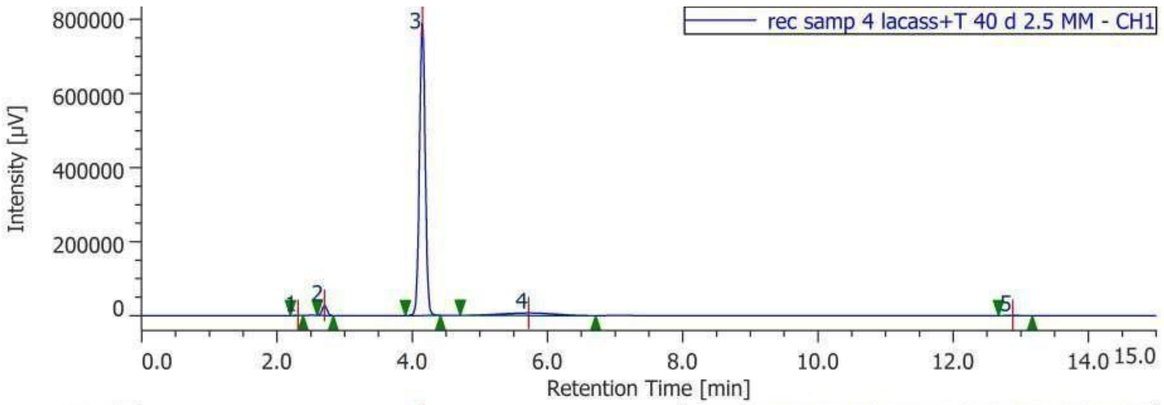
rec samp 4 lacass+T 40 d 2.5 MM - CH1
Intensity [µV]
Retention Time [min]

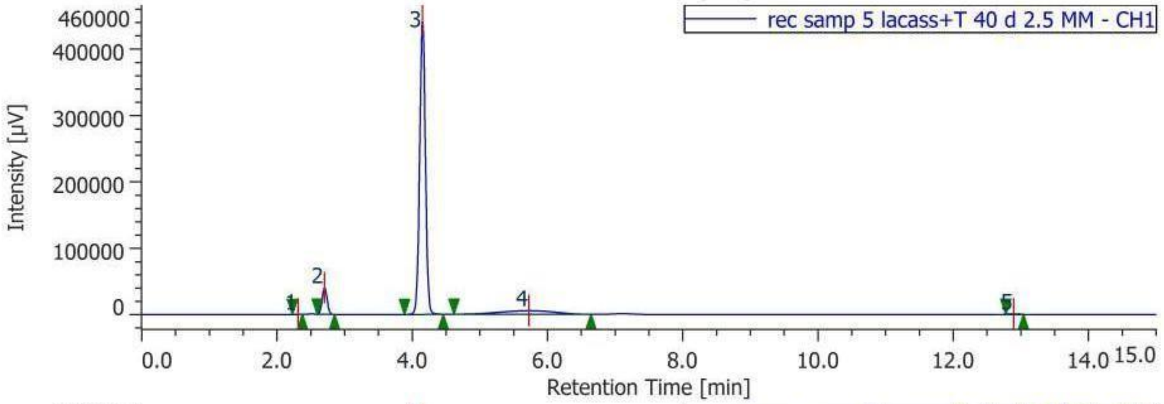
rec samp 5 lacass+T 40 d 2.5 MM - CH1
Intensity [µV]
Retention Time [min]

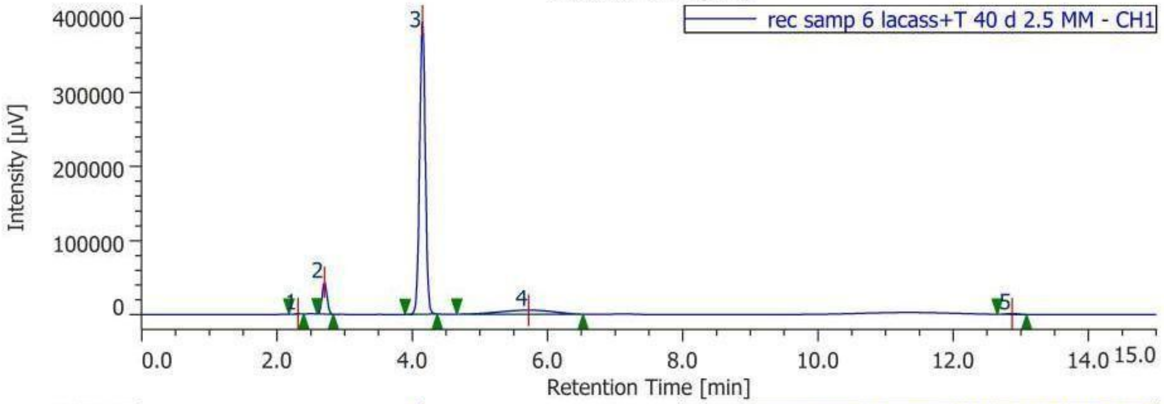
rec samp 6 lacass+T 40 d 2.5 MM - CH1
Intensity [µV]
Retention Time [min]

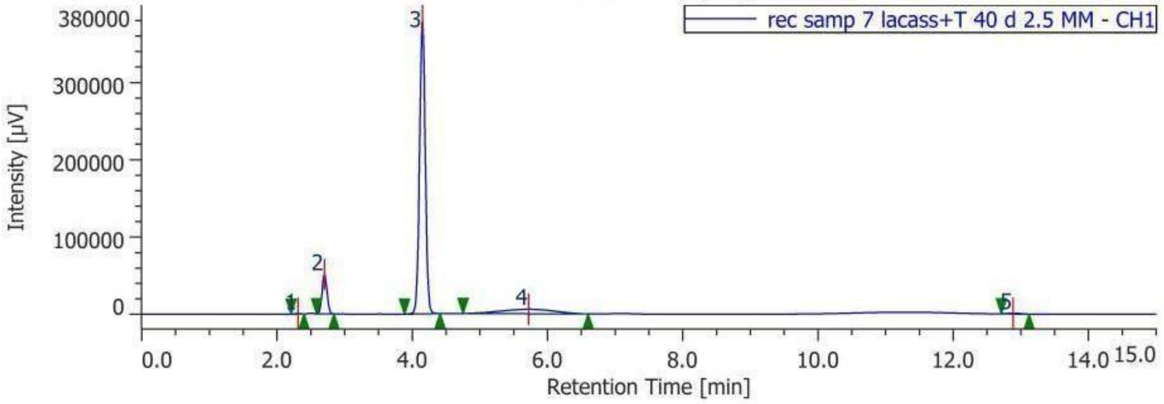
rec samp 7 lacass+T 40 d 2.5 MM - CH1
Intensity [µV]
Retention Time [min]

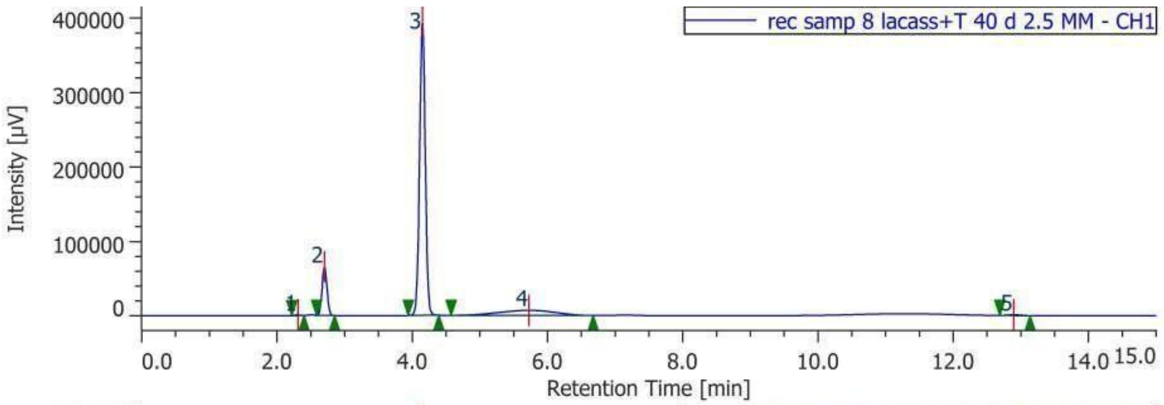
rec samp 8 lacass+T 40 d 2.5 MM - CH1
Intensity [µV]
400000
300000
200000
100000
0
Retention Time [min]
0.0
2.0
4.0
6.0
8.0
10.0
12.0
14.0
15.0

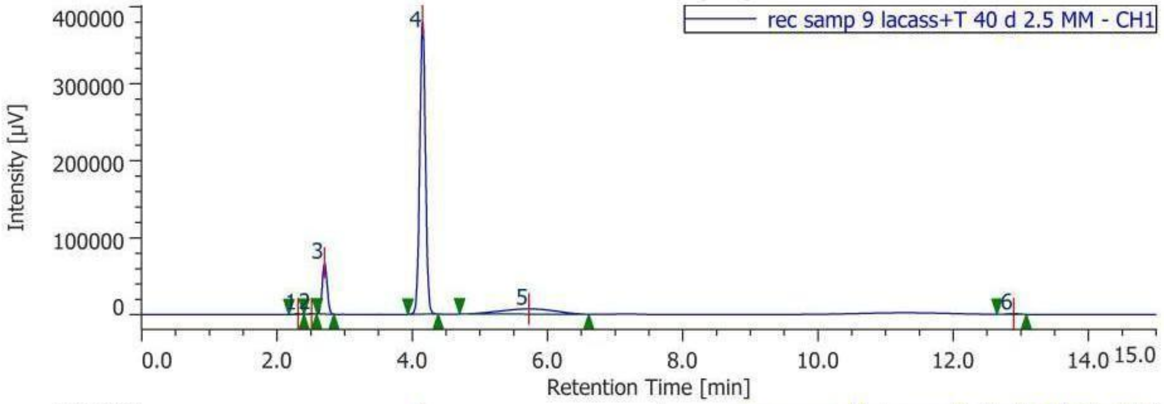
rec samp 9 lacass+T 40 d 2.5 MM - CH1
Intensity [µV]
400000
300000
200000
100000
0
Retention Time [min]
0.0
2.0
4.0
6.0
8.0
10.0
12.0
14.0
15.0

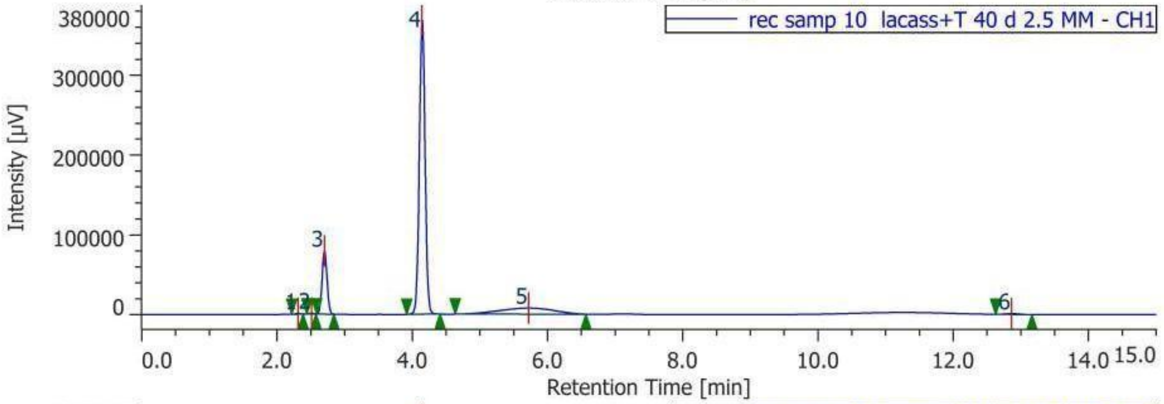
rec samp 10 lacass+T 40 d 2.5 MM - CH1
Intensity [µV]
380000
300000
200000
100000
0
Retention Time [min]
0.0
2.0
4.0
6.0
8.0
10.0
12.0
14.0
15.0

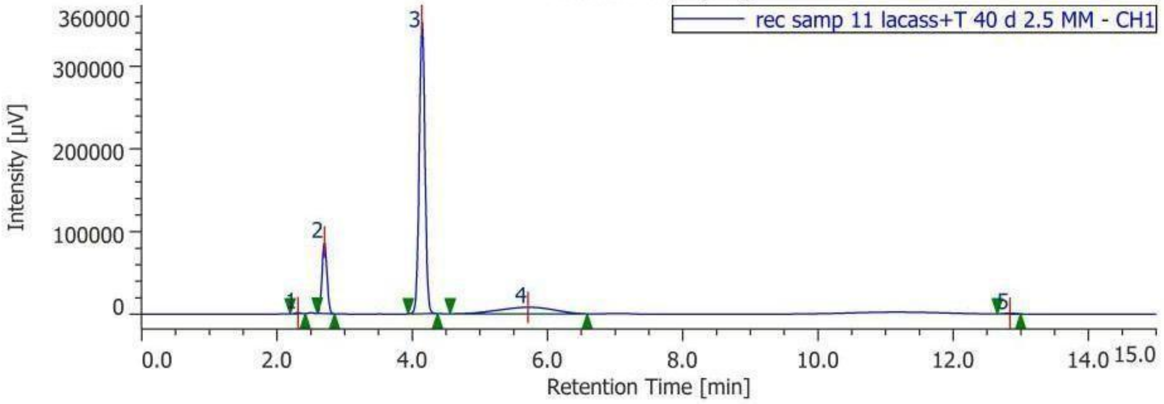
rec samp 11 lacass+T 40 d 2.5 MM - CH1
Intensity [µV]
360000
300000
200000
100000
0
Retention Time [min]
0.0
2.0
4.0
6.0
8.0
10.0
12.0
14.0
15.0

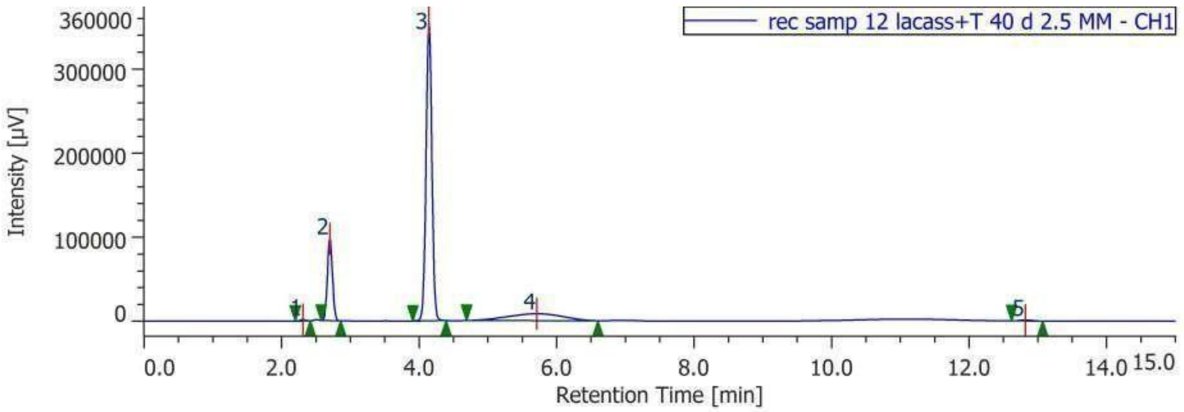

Figure 4: HPLC Chromatogram Showing Formyl Furan Carboxylic Acid Peaks from 0 h to 12h for 25 to 250 mM Substrate Concentrations (1-FDCA, 2-HFCA, 3-HMF, 4-DFF, 5-Phenol [Internal Standard])

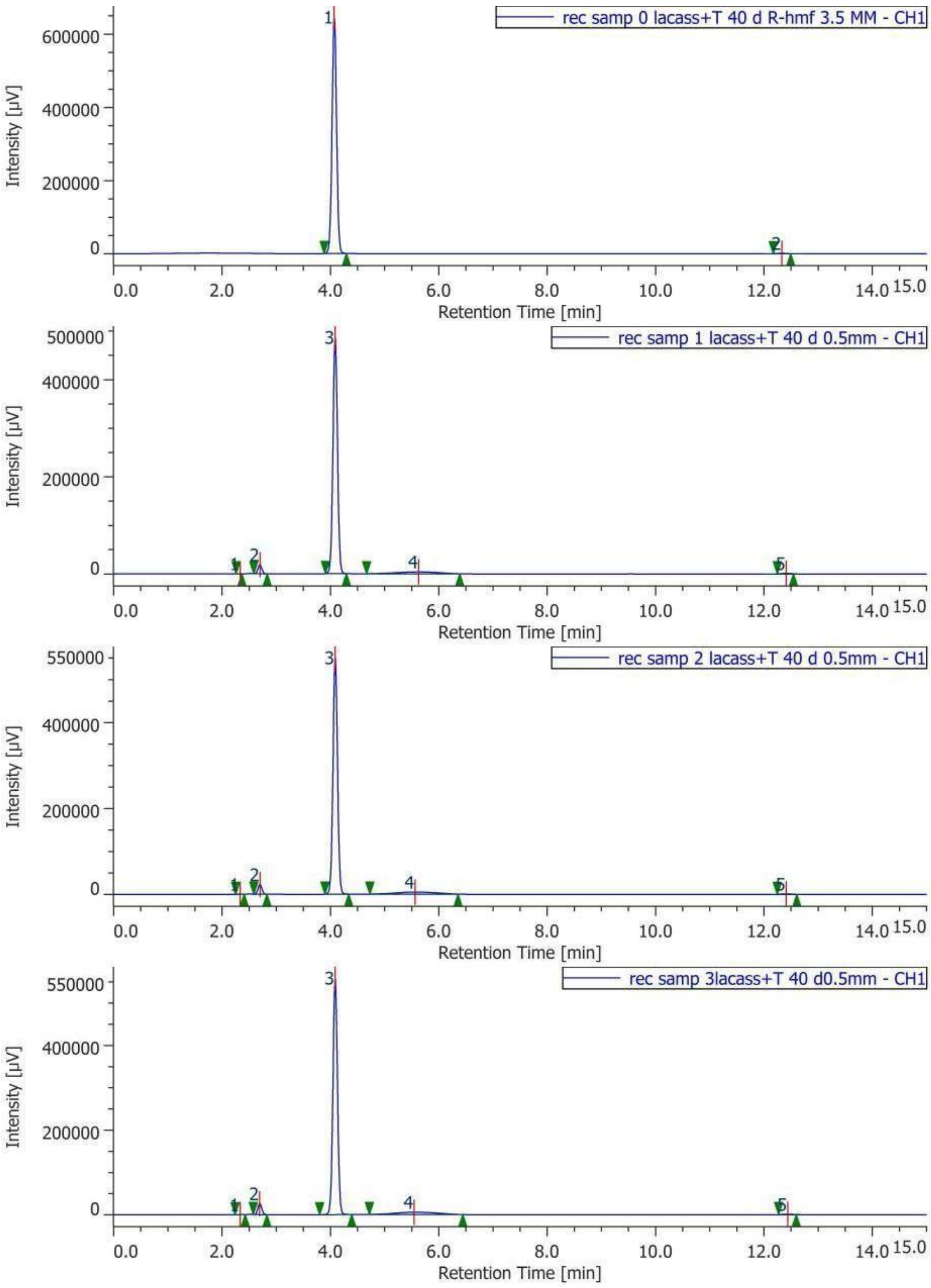
rec samp 0 lacass+T 40 d R-hmf 3.5 MM - CH1
rec samp 1 lacass+T 40 d 0.5mm - CH1
rec samp 2 lacass+T 40 d 0.5mm - CH1
rec samp 3lacass+T 40 d0.5mm - CH1
Intensity [µV]
Retention Time [min]

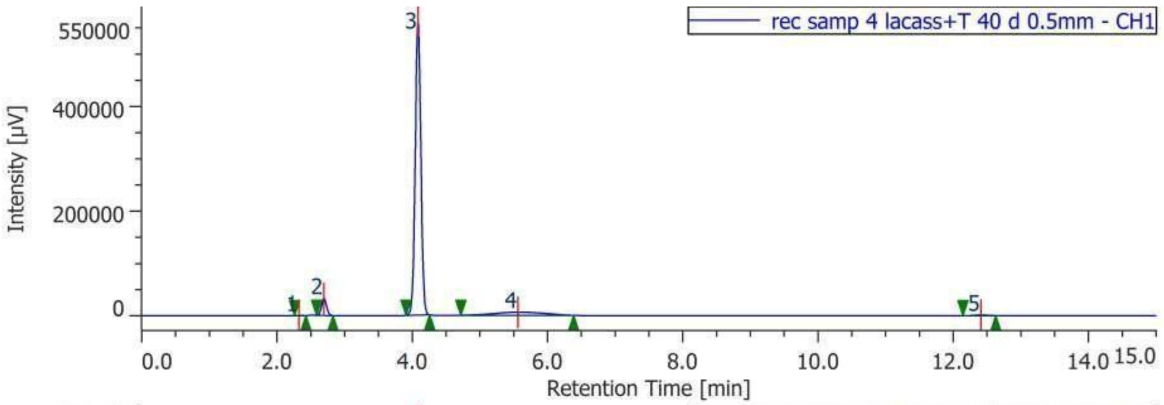
rec samp 4 lacass+T 40 d 0.5mm - CH1
Intensity [µV]
Retention Time [min]

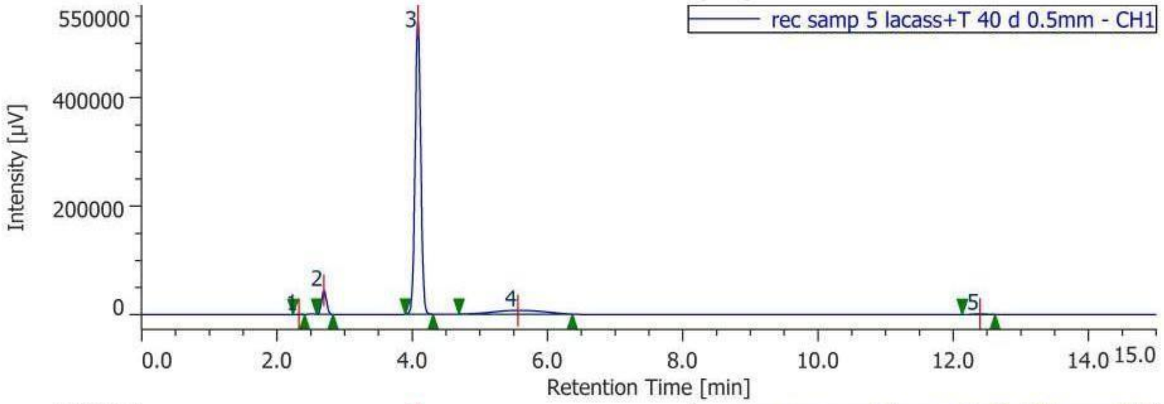
rec samp 5 lacass+T 40 d 0.5mm - CH1
Intensity [µV]
Retention Time [min]

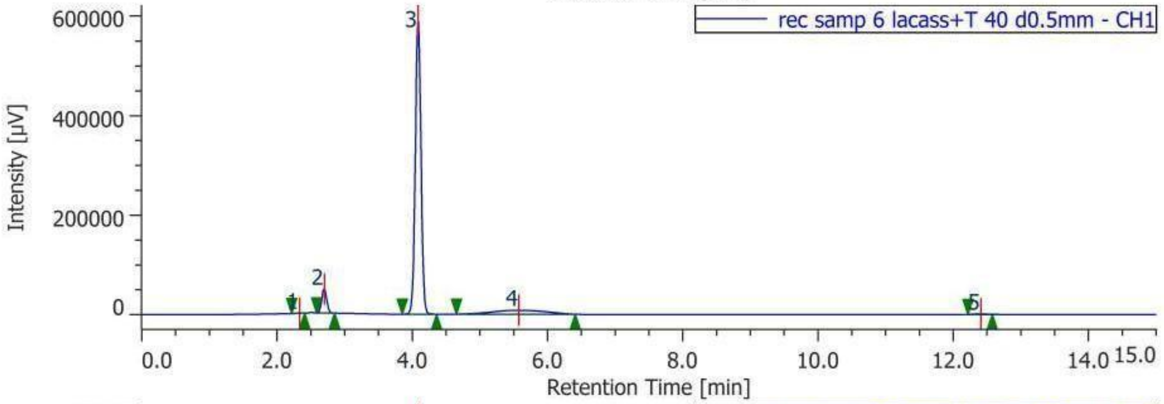
rec samp 6 lacass+T 40 d0.5mm - CH1
Intensity [µV]
Retention Time [min]

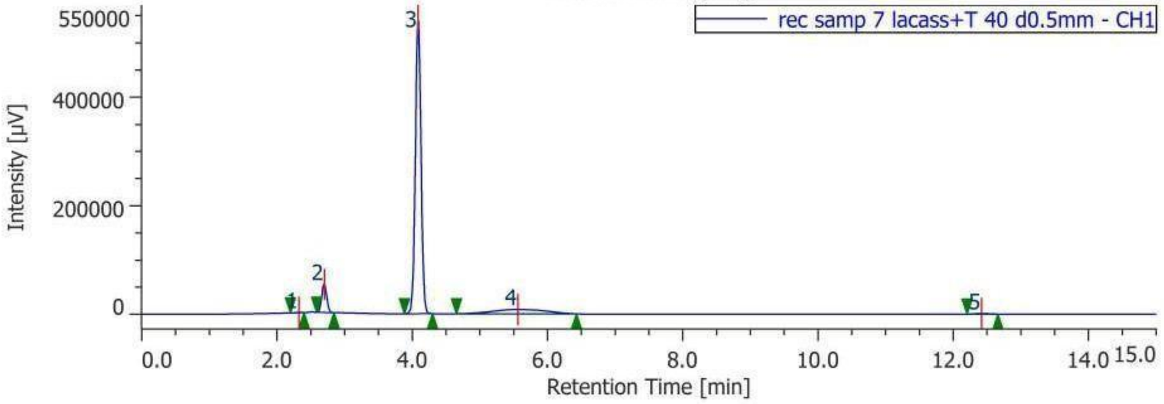
rec samp 7 lacass+T 40 d0.5mm - CH1
Intensity [µV]
Retention Time [min]

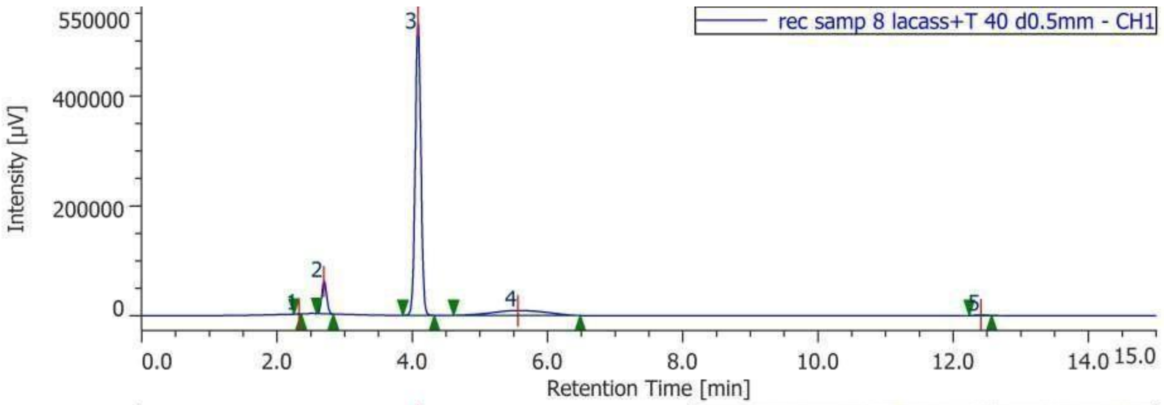
rec samp 8 lacass+T 40 d0.5mm - CH1
Intensity [µV]
Retention Time [min]

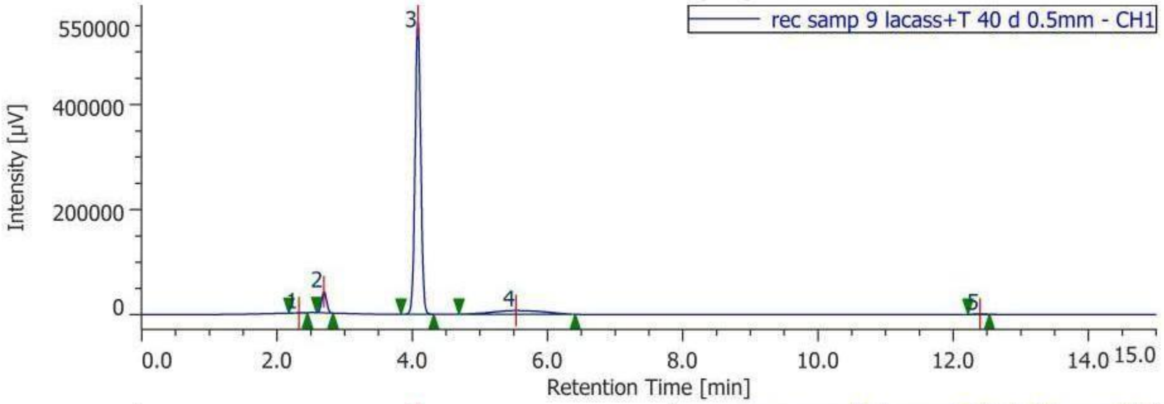
rec samp 9 lacass+T 40 d 0.5mm - CH1
Intensity [µV]
Retention Time [min]

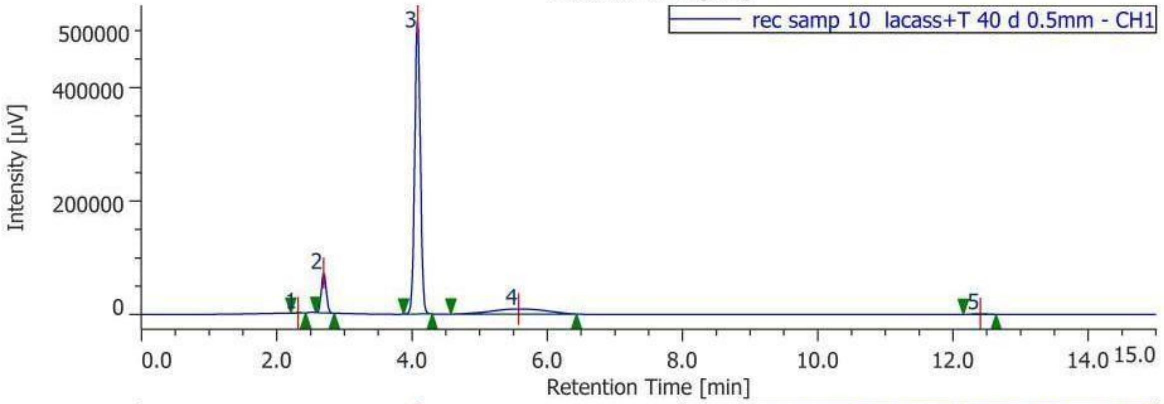
rec samp 10 lacass+T 40 d 0.5mm - CH1
Intensity [µV]
Retention Time [min]

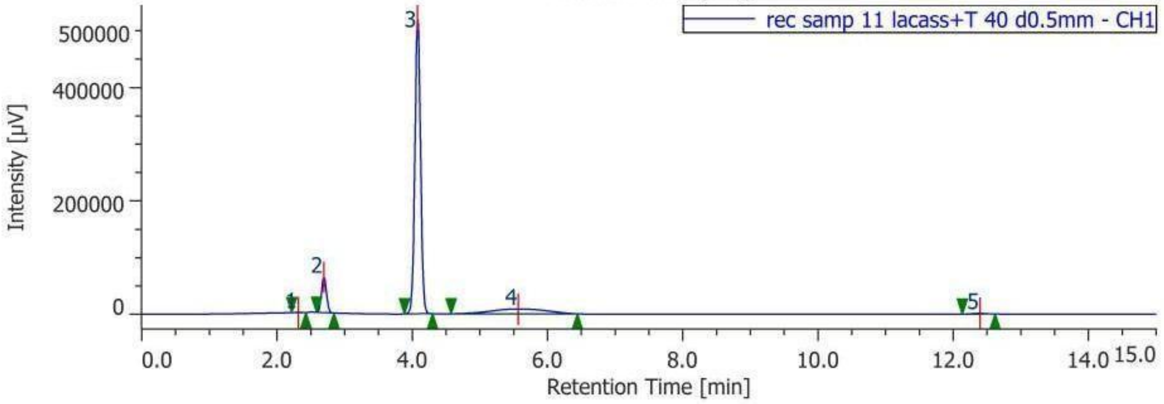
rec samp 11 lacass+T 40 d0.5mm - CH1
Intensity [µV]
Retention Time [min]

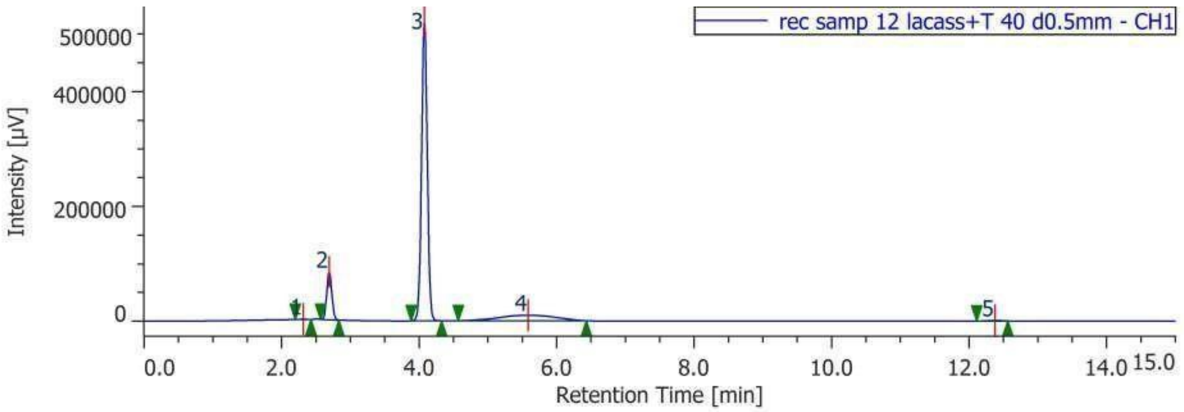

Figure 5: HPLC Chromatogram Showing 2,5-Furan Dicarboxylic Acid Peaks from 0 h to 12h for 25 to 250 mM Substrate Concentrations (1-FDCA, 2-HFCA, 3-HMF, 4-DFF, 5-Phenol [Internal Standard])

Selective Oxidation of Laboratory-Derived 5-HMF to FDCA Under Batch Conditions

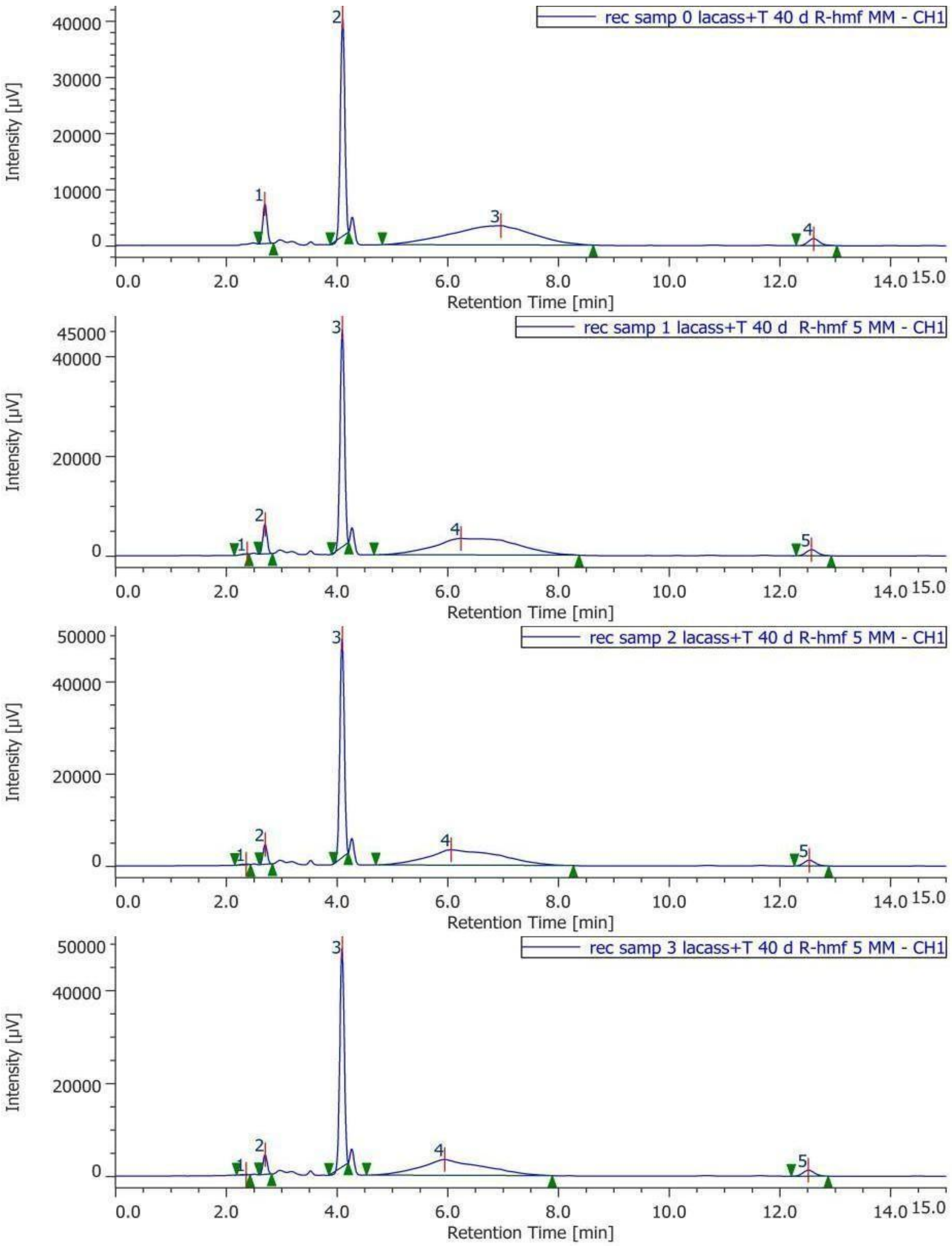

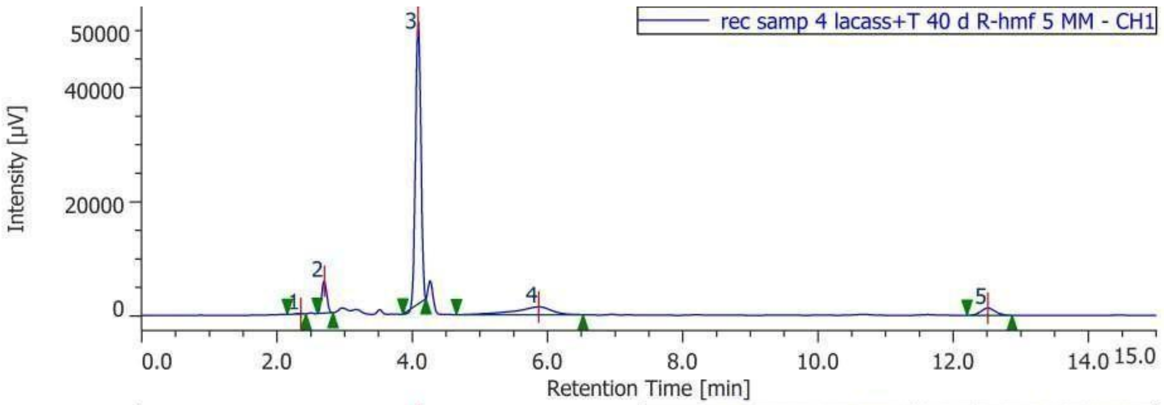
rec samp 4 lacass+T 40 d R-hmf 5 MM - CH1
Intensity [µV]
50000
40000
20000
0
1
2
3
4
5
0.0
2.0
4.0
6.0
8.0
10.0
12.0
14.0
15.0
Retention Time [min]

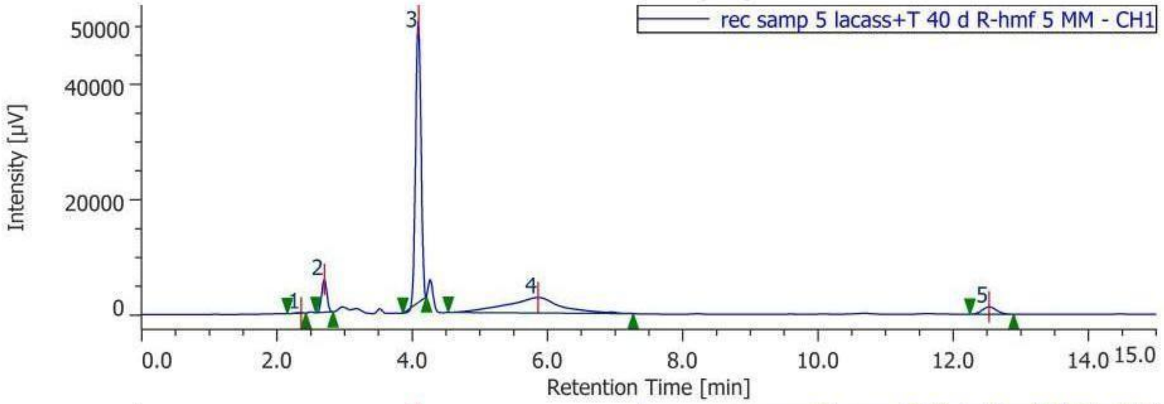
rec samp 5 lacass+T 40 d R-hmf 5 MM - CH1
Intensity [µV]
50000
40000
20000
0
1
2
3
4
5
0.0
2.0
4.0
6.0
8.0
10.0
12.0
14.0
15.0
Retention Time [min]

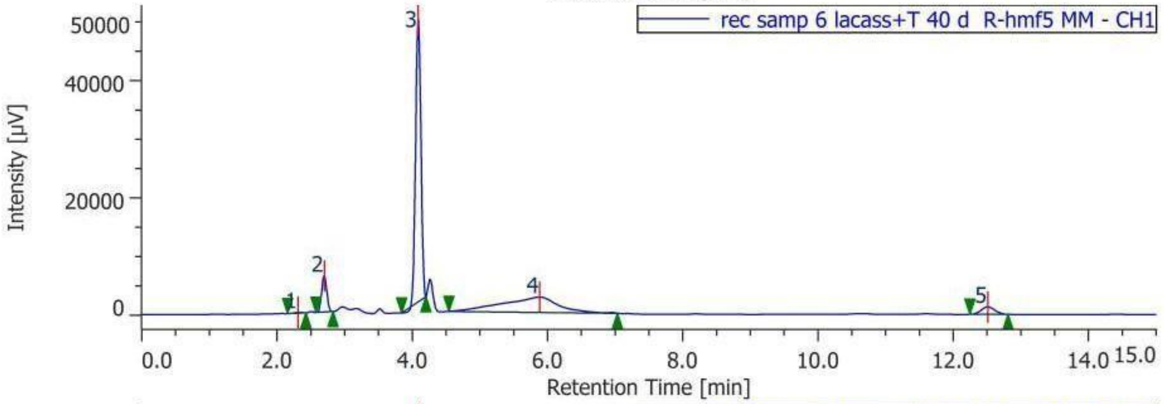
rec samp 6 lacass+T 40 d R-hmf5 MM - CH1
Intensity [µV]
50000
40000
20000
0
1
2
3
4
5
0.0
2.0
4.0
6.0
8.0
10.0
12.0
14.0
15.0
Retention Time [min]

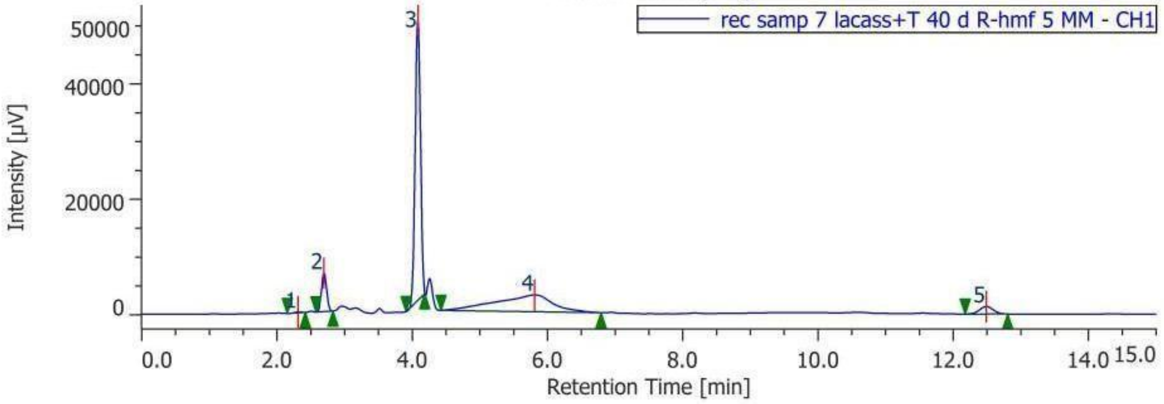
rec samp 7 lacass+T 40 d R-hmf 5 MM - CH1
Intensity [µV]
50000
40000
20000
0
1
2
3
4
5
0.0
2.0
4.0
6.0
8.0
10.0
12.0
14.0
15.0
Retention Time [min]

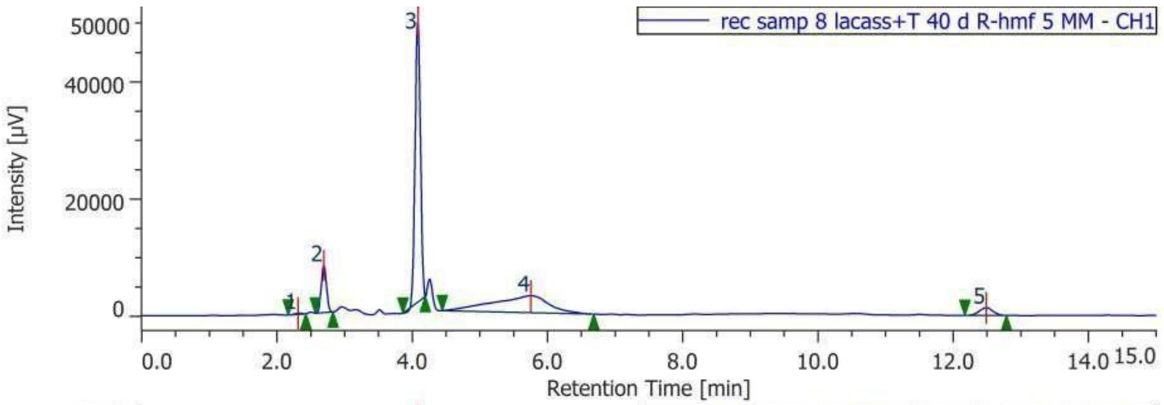
rec samp 8 lacass+T 40 d R-hmf 5 MM - CH1
Intensity [µV]
50000
40000
20000
0
1
2
3
4
5
0.0
2.0
4.0
6.0
8.0
10.0
12.0
14.0
15.0
Retention Time [min]

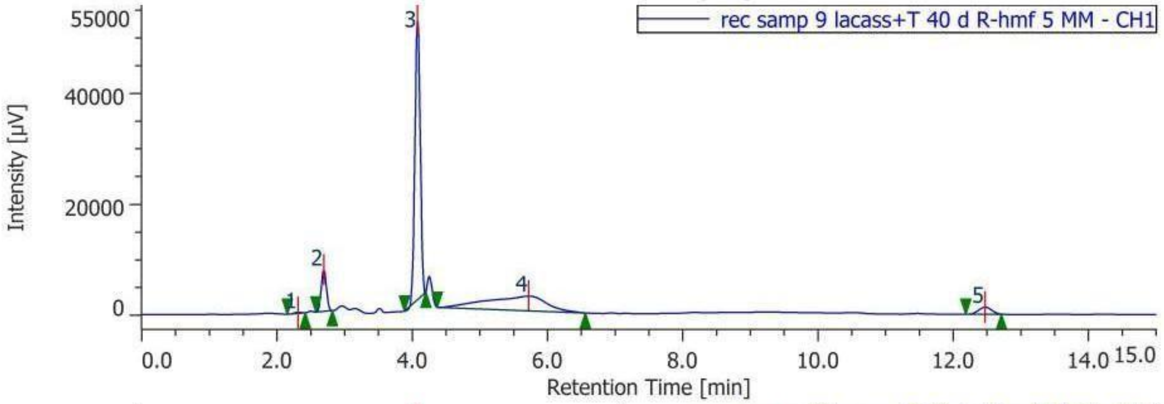
rec samp 9 lacass+T 40 d R-hmf 5 MM - CH1
Intensity [µV]
55000
40000
20000
0
1
2
3
4
5
0.0
2.0
4.0
6.0
8.0
10.0
12.0
14.0
15.0
Retention Time [min]

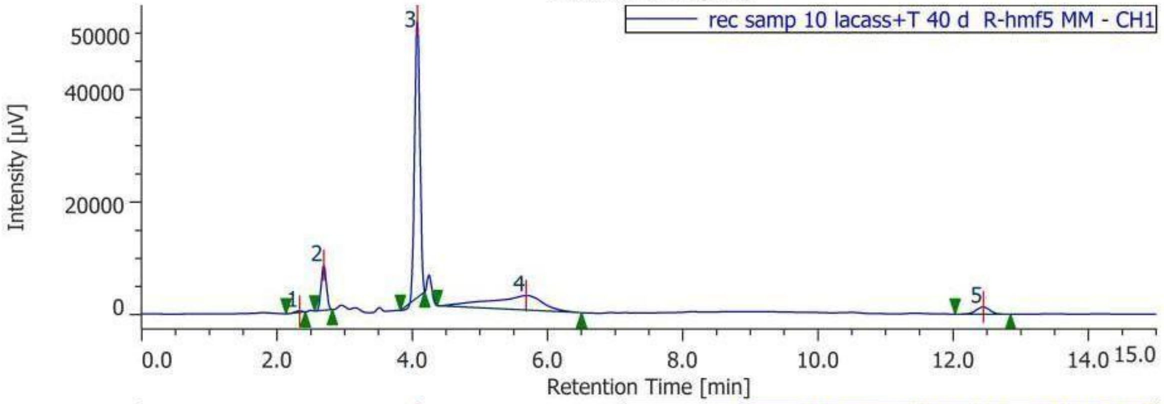
rec samp 10 lacass+T 40 d R-hmf5 MM - CH1
Intensity [µV]
50000
40000
20000
0
1
2
3
4
5
0.0
2.0
4.0
6.0
8.0
10.0
12.0
14.0
15.0
Retention Time [min]

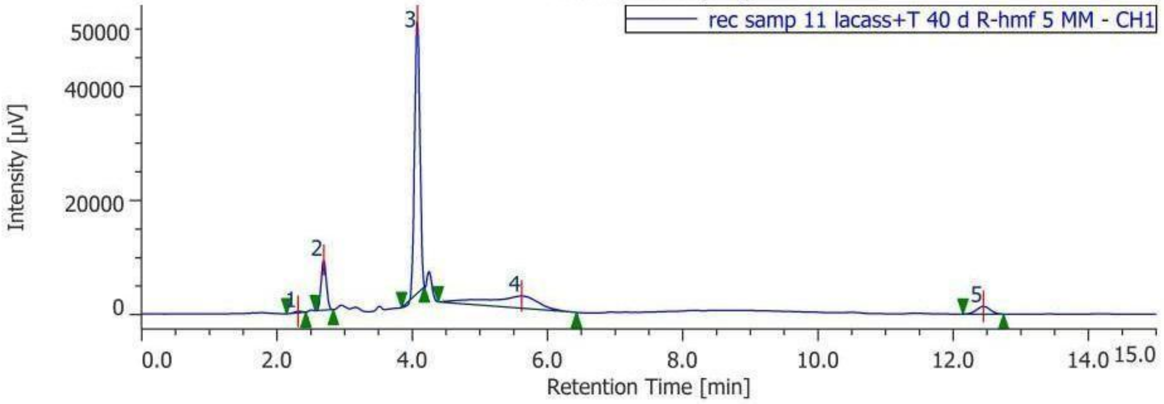
rec samp 11 lacass+T 40 d R-hmf 5 MM - CH1
Intensity [µV]
50000
40000
20000
0
1
2
3
4
5
0.0
2.0
4.0
6.0
8.0
10.0
12.0
14.0
15.0
Retention Time [min]

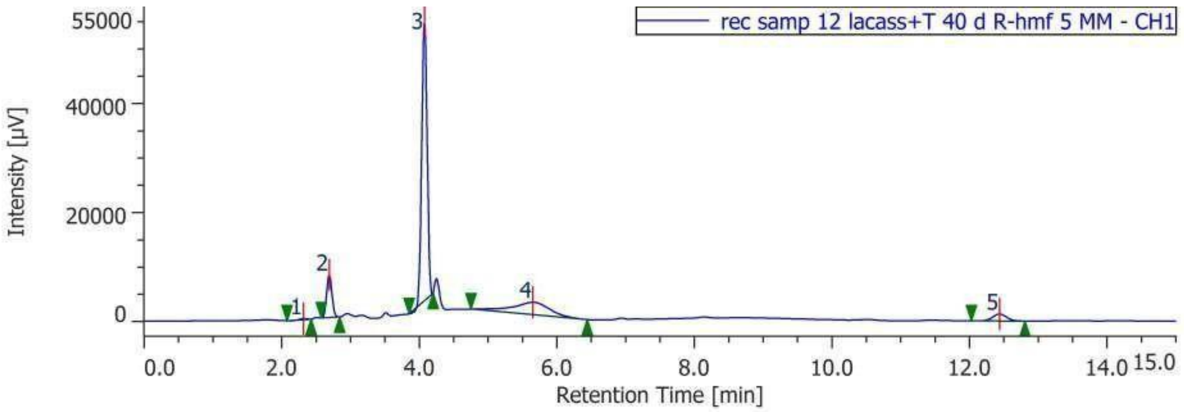

Figure 6: HPLC Chromatogram Showing FDCA, FFCA, DFF, and 5-HMF Peaks with respect to time from 0 h to 12h (1-FDCA, 2-HFCA, 3-HMF, 4-DFF, 5-Phenol [Internal Standard])

Table 1: FTIR data of glass beads treated with APTES (3-Aminopropyltriethoxysilane)

Sr. No.	Structure	Wavelength	Normal Range
**1**	C-N	975.804	800-1600
**2**	C-O	1540	800-1600
**3**	C=O	1698	1600-1800
**4**	O-H	3648	2500-4000
**5**	N-H	3734	2500-4000

Table 2: FTIR data of glass beads treated with APTES (3-Aminopropyltriethoxysilane)

Sr. No.	Structure	Wavelength	Normal Range
1	C-C	1012	800-1600
2	C-O	1456	800-1600
